# Structural, dynamical, and photochemical properties of *ortho*-tetrafluoroazobenzene inside a flexible MOF under visible light irradiation†

**DOI:** 10.1039/d0ra10500g

**Published:** 2021-01-22

**Authors:** Markus Rödl, Samuel Kerschbaumer, Holger Kopacka, Laura Blaser, Felix R. S. Purtscher, Hubert Huppertz, Thomas S. Hofer, Heidi A. Schwartz

**Affiliations:** Institute of General, Inorganic and Theoretical Chemistry, University of Innsbruck Innrain 80-82 A-6020 Innsbruck Austria heidi.schwartz@uibk.ac.at t.hofer@uibk.ac.at

## Abstract

Considering porous materials as host matrices is an elegant way to enable photoswitching of non-covalently attached organic dyes even in the solid state. By focusing on the resulting optical properties as a function of loading degree and synthesis procedure, the occurring host–guest and guest–guest interactions can be determined and further exploited. In the course of this study, the photochromic behavior of *ortho*-tetrafluoroazobenzene (tF-AZB) inside flexible DMOF-1 was investigated from these points of view. It was found that depending on the loading degree and temperature, tF-AZB shows varying *E*/*Z* ratios and switching efficiency. For systems with low loading, reversible visible light induced isomerization was observed over ten switching cycles: Upon violet light exposure, formation of 100% *E* isomer was generated, while green light irradiation resulted in ∼60% *Z*-tF-AZB. Complementary molecular dynamics simulations at DFTB (density functional tight binding)-level revealed changing binding sites for *Z*-tF-AZB inside DMOF-1. For the *E* isomer, only low oscillations have been found, which in turn display a rare T-stacking interaction. Although the interaction strengths of the *E* and *Z* isomers with DMOF-1 are in the same range, the different mobility of both isomers due to varying binding sites explains the preference of the *E* isomer even upon green light exposure.

## Abstract 2

Understanding isomer distribution of tF-AZB inside DMOF-1 as a function of host–guest and guest–guest interaction.

## Graphical abstract

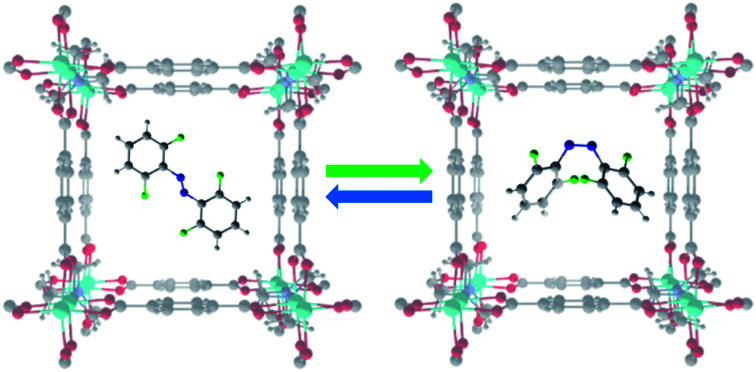

## Introduction

Several recent findings on the embedment of photoactive dye molecules into host matrices such as polymers^1–5^ or nanoporous materials^6–14^ have led to great motivation behind this field of research. In fact, the combination of these molecules with an amorphous or crystalline material was found to be profitable, as the interactions can result in enhanced or even new functions.^15^

**Fig. 1:**
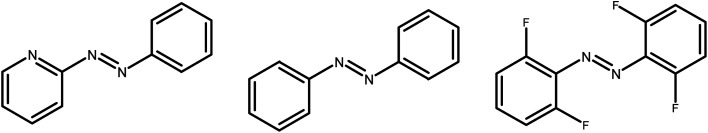
Structures of the *E* isomers of 2-(phenylazo)pyridine, azobenzene, and *ortho*-tetrafluoroazobenzene.

Starting with the work of Fujita in 2010,^16^ metal–organic frameworks (MOFs) were firstly applied as porous crystalline host matrices for a photoswitchable dye molecule. Hereby, solid state switching of a stilbene was observed inside this class of porous host materials. MOFs are hybrid materials consisting of organic linker molecules and inorganic nodes that form 3 D networks with potential voids.^17^ Besides their diverse applicabilities regarding *e.g.* gas adsorption,^18,19^ separation of gases^20,21^ and liquids,^21–23^ drug encapsulation and delivery,^24,25^ incorporation of fluorescent dyes,^26,27^ photoconductivity,^28^ or implementation in electronic devices,^29,30^ this substance class has gained increasing attention in photoactive guest encapsulation.^31–35^ Particularly, it was focused on the incorporation of the photochromic moiety as non-covalently attached guest molecules with stilbenes,^16^ 2-(phenylazo)pyridine (denoted as PAP in the following),^36,37^ azobenzenes and (fluorinated) derivatives (denoted as (xF-)AZB),^38–43^ diarylethenes,^44^ spiropyrans,^45–48^ or spirooxazines.^49^ Several functional materials with promising properties were obtained, *e.g.* photoswitching conductance^47^ or guest-to-host transmission of structural changes.^42^ Especially, the latter showed an impressive way of how light information can be converted into a mechanical one. Kitagawa and co-workers^42^ embedded a non-substituted azobenzene into the flexible MOF DMOF-1.^50^ Irradiation with UV light resulted not only in the *E*-to-*Z* conversion (photostationary state of an *E*/*Z* ratio of 62 : 38) of the guest molecule, but also in structural changes of the host material itself. This structural guest-to-host transmission was then used to successfully switch the gas adsorption properties of the AZB@DMOF-1 hybrid material. A few years later, Agarkar and Das^37^ repeated these experiments with the structurally related 2-(phenylazo)pyridine (PAP). The similarity of AZB and PAP is shown in Fig. 1. Surprisingly, the *E*-to-*Z* conversion of PAP did not affect the structure of the DMOF-1 framework at all, even though the *E*/*Z* ratios of 72 : 28 were comparable to those of azobenzene inside DMOF-1.

As a major drawback, the isomerization processes of AZB and PAP are exclusively triggered by UV light, which might interfere with the host matrix and is energetically unfavorable. Furthermore, the absorption bands of the *E* and *Z* isomer of AZB overlap, suppressing a complete transformation of one isomer into the other. An elegant way to avoid these problems is the systematic functionalization of the switchable molecule that leads to the separation of the absorption bands.^51^ By introducing fluorine substituents in the phenyl rings, *e.g. ortho*-tetrafluoroazobenzene (denoted tF-AZB), almost quantitative switching (91% *Z* and 86% *E*) was reached, when dissolved in acetonitrile.^52^ Additionally, these isomerization processes can be triggered by light in the visible region. Hence, UV light is no longer necessary to induce the *E*-to-*Z* transformation.^52^ This is of upmost importance, because UV light naturally has the tendency to degrade materials. The described advantages were exploited by Ruschewitz and co-workers, who employed fluorinated azobenzenes (tF-AZB, 4*H*,4*H*′-octafluoroazobenzene (oF-AZB), and perfluoroazobenzene (pF-AZB)) as guest molecules and embedded them into various MOF host materials.^43^ Remarkably, the authors found nearly quantitative switching for tF-AZB and oF-AZB of up to 100% *E* and *Z*, when incorporated into hosts of the MIL-68 family.^53^ In this work, tF-AZB was embedded into flexible DMOF-1. The resulting hybrid systems are expected to exhibit high switching yields, which, in turn, would allow for an effective way to structurally influence the host lattice by visible light irradiation. Furthermore, the optical properties were investigated as a function of loading degree and synthesis temperature. For the former, three dilutions were chosen in order to (a) determine the maximum loading degree and to (b) study the photochromic properties with respect to possible guest–guest and, by inserting only one tF-AZB molecule in a 2·2·2 supercell of DMOF-1, exclusively host–guest interactions.

For the latter, the impact of two synthesis temperatures on the presence of *E* and *Z* isomers in the initial and irradiated state was investigated. Azobenzenes belong to the T-type chromophores, which are known to reconvert to their thermodynamically stable state under heat supply.^54^ As a consequence, we expect the higher synthesis temperature to significantly impact the *E*-to-*Z* ratio of the non-irradiated compounds.

In order to provide a detailed analysis of the guest–host interaction, theoretical calculations have been performed. The focus was set on the determination of the preferred interaction site of a single tF-AZB molecule inside the DMOF-1 structure. In order to study the system at an elevated temperature (*i.e.* 298 K), a molecular dynamics (MD) simulation approach was chosen over a simple analysis based on an energy minimization representing the system only at 0 K. Taking into account that the *E* form of tF-AZB could potentially bind to two adjacent BDC^2−^ residues, a simulation system considering a 2·2·2 supercell, *i.e.* a [Zn_2_(BDC)_2_(DABCO)]_8_ system, was used to reduce the impact of guest–guest interactions under periodic boundary conditions. This comparably large system size containing a total of 2320 electrons already proves too demanding for a picosecond-scale molecular dynamics study employing density functional theory (DFT). However, the increasingly successful density functional tight binding (DFTB) approach,^55,56^ representing a DFT-parametrized variant of tight binding (TB) theory,^57^ proved as a viable alternative to balance the accuracy of results and the associated computational demand. In this work, self-consistent charge density functional tight binding (SCC DFTB)^58,59^ interfaced to our in-house developed molecular dynamics simulation engine^60–62^ was applied to study the properties of the *E* and *Z* isomers of tF-AZB in the DMOF-1 host lattice.

## Results

The synthesis of the tF-AZB@DMOF-1 systems was performed *via* a gas phase loading process to exclude any solvent molecules from all further considerations. This procedure has been successfully conducted in prior works on azobenzenes,^38,40,43^ spiropyrans,^45,47^ and spirooxazines.^49^ Further incorporation methods include diffusion,^16,40,47,63^ melting of the dye molecule to enter the MOF pores,^44^ or crystallization inclusion during the host synthesis procedure.^46^ Notably, several studies have been published on MOFs with the photoswitchable molecule being either substituent of the linker^64–72^ or the linker backbone^73–75^ itself. Additionally, the photochromic dye can be immobilized *via* coordinative bonds.^72^ We chose an excess of tF-AZB (molar ratio guest-to-host of 3 : 1), an equal molar ratio, and a high dilution to study possible influences of the loading degree on the optical properties of the initial and the irradiated species. In order to take possible thermal impact into account, the synthesis of the 1 : 1 compound and the highly diluted compound (one molecule tF-AZB in a 2·2·2 supercell DMOF-1) was performed at two different temperatures. That way, five hybrid systems were obtained, which are listed in the following Table 1.

**Table 1:** List of the obtained compounds with the respective starting molar ratio and synthesis temperature

	System 1	System 2	System 3	System 4	System 5
Molar ratio (guest : host)	3 : 1	1 : 1	1 : 1	0.125 : 1	0.125 : 1
Maximum synthesis temperature	70 °C	65 °C	105 °C	65 °C	105 °C

These hybrid systems were analyzed following the protocol of the ICE-principle, regarding successful incorporation, composition, and resulting optical properties and possible guest-dependent structural transformations (effects).^49^ It has to be stated that the synthesis as well as the analysis of the resulting system was exclusively performed in the dark to avoid any influence of daylight on the isomer ratios in the non-irradiated and irradiated species.

### Analysis of successful incorporation and composition

**Fig. 2:**
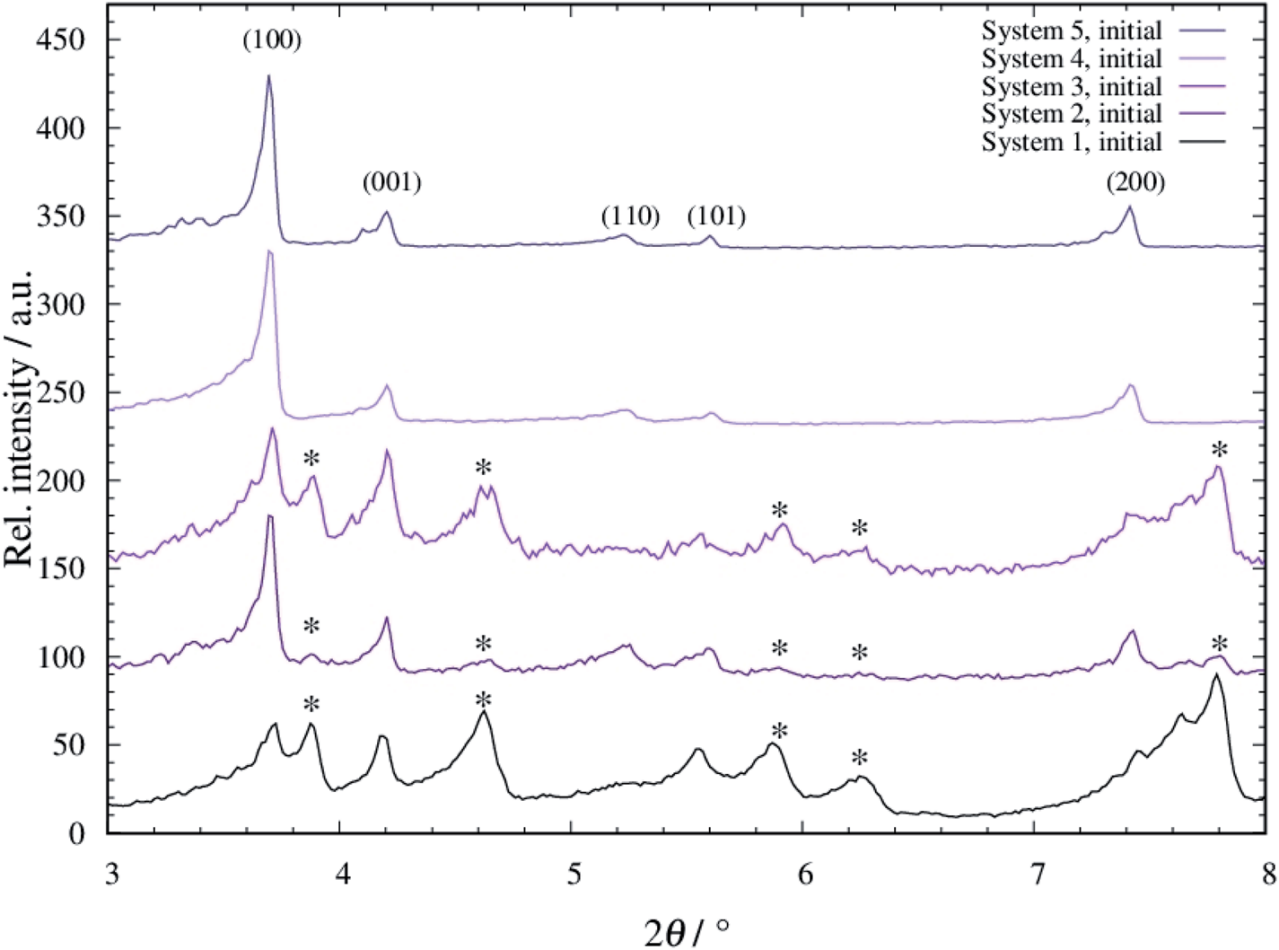
Diffraction patterns of tF-AZB@DMOF-1 systems 1 to 5. The diffraction patterns were measured at 298 K (Stoe Stadi P: *λ* = 0.7093 Å). New reflections occurring after the dye embedment are marked with an asterisk.

For all systems 1 to 5, tF-AZB was successfully incorporated into DMOF-1. The respective diffraction patterns are shown in Fig. S1 to S5, ESI.† When comparing the diffraction patterns of the composite materials to the single components, the main reflections of the host material remain, as visible in the unchanged 2*θ* values. However, new reflections occur. These additional reflections do not originate from free tF-AZB, as its main characteristics at 2*θ* values of 11°–13° are not present. Conclusively, tF-AZB has been successfully incorporated into DMOF-1 for all systems 1 to 5. In Fig. 2, the XRPD patterns of all systems 1 to 5 are compared.

In all patterns, the reflections originating from DMOF-1 are present (see also Fig. S1 to S5, ESI†). Furthermore, additional reflections occur (marked with an asterisk). These reflections are sharply formed for systems 1 and 3, slightly present for system 2 and not found for systems 4 and 5. By comparing the obtained data to unloaded DMOF-1 and benzene-loaded DMOF-1,^50^ these additional reflections originate from an orthorhombic cell. Therewith, systems 1 to 3 appear as a superposition of tetragonal and orthorhombic reflections, which has also been found by Kitagawa and co-workers^42^ and Agarkar and Das^37^ (see Fig. S6, ESI†). Interestingly, these orthorhombic reflections do not or only weakly occur at low loading with tF-AZB (systems 4 and 5) and at comparatively low synthesis temperatures (system 2). Therefore, the superposition of tetragonal and orthorhombic reflections is assumed to be the result of host–guest interactions between the MOF scaffold and the embedded dye molecule, which are significantly influenced by the amount of tF-AZB (and possible guest–guest interactions as well as isomer ratios) and synthesis temperature.

To understand the occurring effects, liquid-state NMR spectroscopic measurements were performed to determine the amount of embedded dye and the amount of *E* isomer being present in the initial state. In Table 2, the results of these measurements are listed. The respective spectra can be found in Fig. S7 to S11, ESI.†

**Table 2:** Results of the liquid-state NMR spectroscopic investigations on systems 1 to 5 in the initial state

	System 1	System 2	System 3	System 4	System 5
Composition (tF-AZB : DMOF-1)	1.5 : 1	1 : 1	0.9 : 1	0.125 : 1	0.11 : 1
*E* isomer	88.7%	72.4%	**100%**	**100%**	**100%**
*Z* isomer	11.3%	27.6%	0%	0%	0%

For the synthesis of system 1, an excess of tF-AZB was applied to determine the maximum loading, which is 1.5 tF-AZB per formula unit DMOF-1 (see Table 2 and Fig. S11, ESI†). The composition of 2 to 5 reflect the molar ratios used for the synthesis. A comparison of the isomer ratios shows that the systems with low loading (systems 4 and 5) and high synthesis temperature (system 3) are at 100% *E*, whereas only 88.7% and 72.4% are found for systems 1 and 2. Conclusively, exclusive host–guest interactions (for low loading amounts) as well as heat supply cause T-type tF-AZB to be present in its *E* form.

### Optical properties of the tF-AZB@DMOF-1 hybrid materials

To study and track the photochromic behavior of tF-AZB inside DMOF-1, UV/Vis reflectance spectra before and after irradiation (*λ* = 405 nm and *λ* = 535 nm) were collected. In solution, exposure to electromagnetic radiation with a wavelength of *λ* = 405 nm causes the formation of the *E* isomer, while green light (*λ* = 530 nm) irradiation results in the respective *Z* isomer. For tF-AZB, the *Z* isomer shows a characteristic absorption maximum at 414 nm for the n–π* transition.^76^ The n–π* transition and π–π* transition of the *E* isomer are located at 458 nm and 305 nm, respectively.^52,76^ In Fig. 4 and 5, the UV/Vis spectroscopic studies on systems 1 to 5 are depicted.

**Fig. 3:**
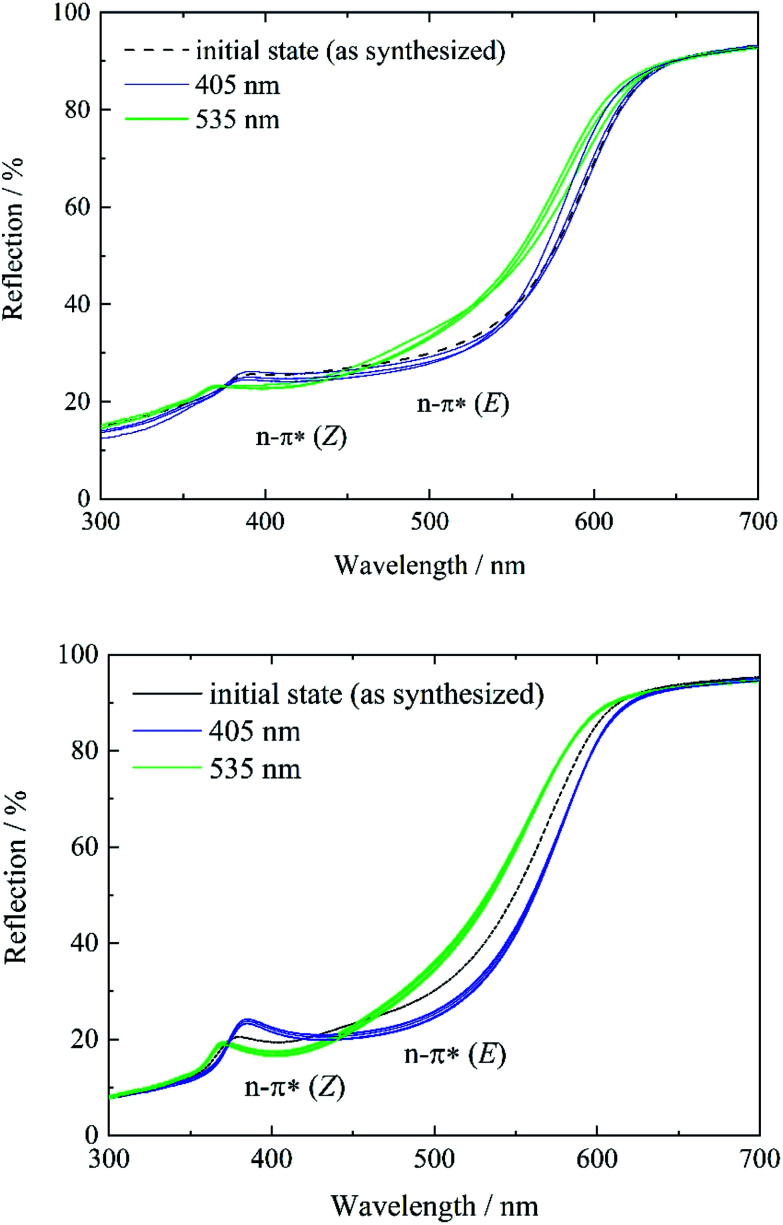
Reflectance spectra of systems 1 (top) and 2 (bottom) before (black) and after irradiation with violet light (*λ* = 405 nm) (blue) and green light (*λ* = 535 nm) (green). For system 1, three, and for system 2, 10 switching cycles were performed to demonstrate the reversibility of the *E*-to-*Z* isomerization.

In the initial state, systems 1 and 2 show characteristics of both the *E* and the *Z* isomer (Fig. 3, left, black lines and Fig. S12, ESI†). This is visible in two reflection minima located at approx. 400 nm and 480 nm (n–π* transition of the *Z* isomer and n–π* transition of the *E* isomer, respectively). Upon irradiation with violet light, the minimum corresponding to the n–π* transition of the *Z* isomer vanishes, while the minimum for the n–π* transition of the *E* isomer is further processed (Fig. 3, blue lines). Therefore, a proceeding *E* isomer formation upon violet light irradiation was concluded. In a next step, the hybrid systems were exposed to green light (*λ* = 535 nm) causing the formation of a minimum at approx. 400 nm, whereas the reflection minimum of the *E* isomer almost disappears. This minimum can be attributed to the n–π* transition of the *Z* isomer. Hence, photoisomerization of tF-AZB inside DMOF-1 is possible. The irradiation experiments were repeated over 10 cycles, which are shown in Fig. 3, right. For system 1, already after three cycles a varying absorption behavior is observed, whereas for system 2, the minima of the respective transitions of the *E* and *Z* isomer are present and formed without any hint of fatigue.

**Fig. 4:**
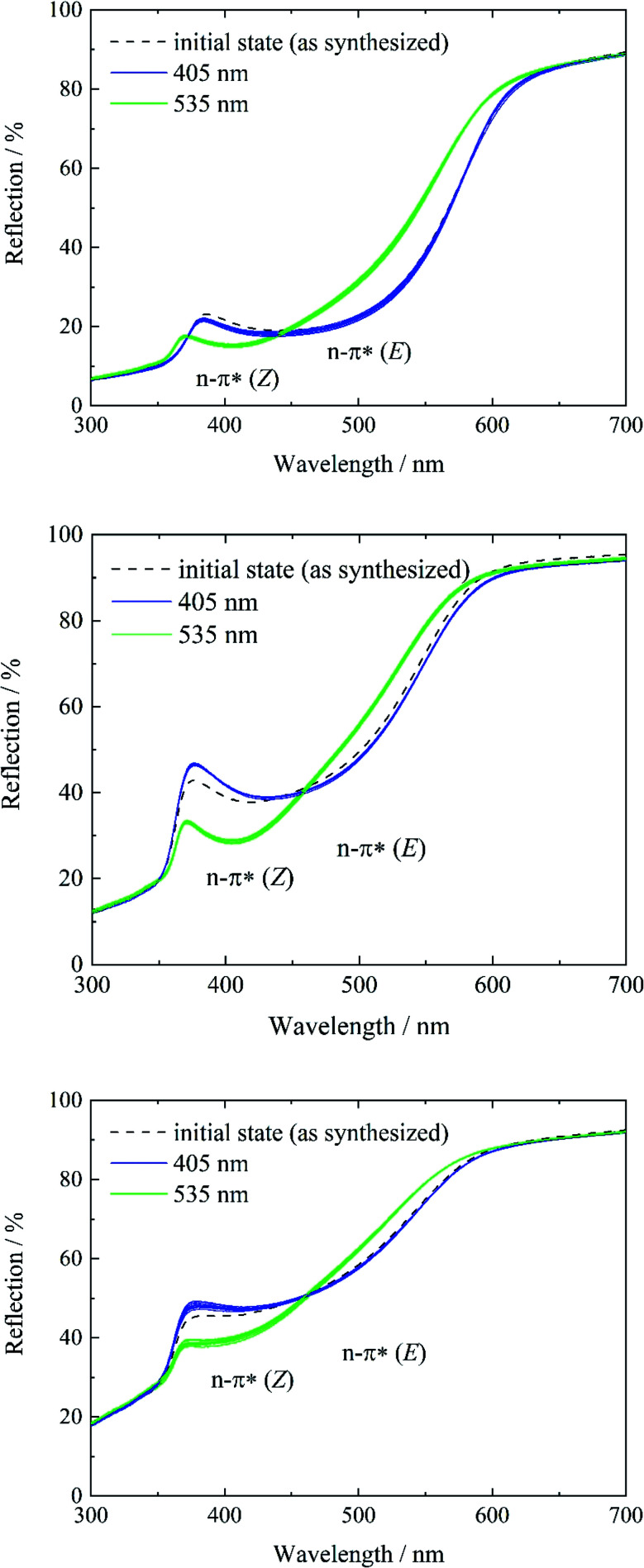
Reflectance spectra of systems 3 (top), 4 (middle), and 5 (bottom) before (black) and after irradiation with violet light (*λ* = 405 nm) (blue) and green light (*λ* = 535 nm) (green). For each system, 10 switching cycles were performed to demonstrate the reversibility of the *E*-to-*Z* isomerization.

In contrast, systems 3 to 5 only show characteristics of the *E* isomer in the initial state (see Fig. S13, ESI†). As a result of the high temperature at the end of the gas phase synthesis (system 3) and the low loading degree (systems 4 and 5), the *E* isomer is predominantly present. Subsequent violet light exposure does almost not lead to any change in the reflection spectrum in comparison to the initial state (Fig. 4, left, blue lines), which points to a 100% *E*-tF-AZB inside DMOF-1, which has already been shown by liquid-state NMR spectroscopy (see Table 2). The *E*-to-*Z* conversion is reversibly triggered by green light (Fig. 4, green lines) and no hint of fatigue is found after ten switching cycles (Fig. 4).

As a result of low loading, guest–guest interactions are reduced, while host–guest interactions must increase. Due to almost any guest–guest interactions, the preferred orientation of the guest molecule inside the host scaffold must be at an energetical minimum, which seems to be reached when tF-AZB is in its *E* form. This is visible for systems 3 to 5 in the initial state (Fig. 4), but not for systems 1 and 2. In order to probe the *E*-to-*Z* ratio after irradiation with violet and green light, respectively, NMR spectroscopic experiments were conducted after irradiation with (a) violet light (*λ* = 405 nm) and (b) green light (*λ* = 535 nm). The obtained results are listed in Table 3, the corresponding NMR spectra can be found in Fig. S12 to S21, ESI.†

**Table 3:** Results of the liquid-state NMR spectroscopic investigations on System 1 to 5 in the initial state

	System 1	System 2	System 3	System 4	System 5
**405 nm, 15 min**					
*E* isomer	93%	94.6%	100%	100%	100%
*Z* isomer	7%	5.4%	0%	0%	0%

**535 nm, 15 min**					
*E* isomer	60.5%	39.3%	37.7%	38%	40%
*Z* isomer	39.5%	**60.7%**	**62.3%**	**62%**	**60%**

In accordance to the obtained results *via* UV/Vis spectroscopy, the formation of the *E* isomer is induced by violet light irradiation. Here, system 1 shows 93% of *E* isomer, whereas even a higher amount is reached for system 2 with 94.6%. As expected from the ratios determined for the initial states before (see Table 2), the amount of *E* isomer does not change for systems 3 to 5. Subsequent irradiation with green light causes the formation of the *Z* form, which has already been followed by UV/Vis spectroscopic investigations. With only approx. 40% of *Z* isomer being generated, system 1 shows the least efficient *E*-to-*Z* conversion. It is assumed that this originates from densely packed tF-AZB inside the MOF pores, which sterically hinders proceeding isomerization. In contrast, ∼60% of *Z* isomer is found for the other systems 2 to 5. For these systems, the spatial freedom enhances the efficiency of isomerization. However, a quantitative switching between both isomers was not achieved.

### Investigation of potential structural guest-to-host transmissions

**Fig. 5:**
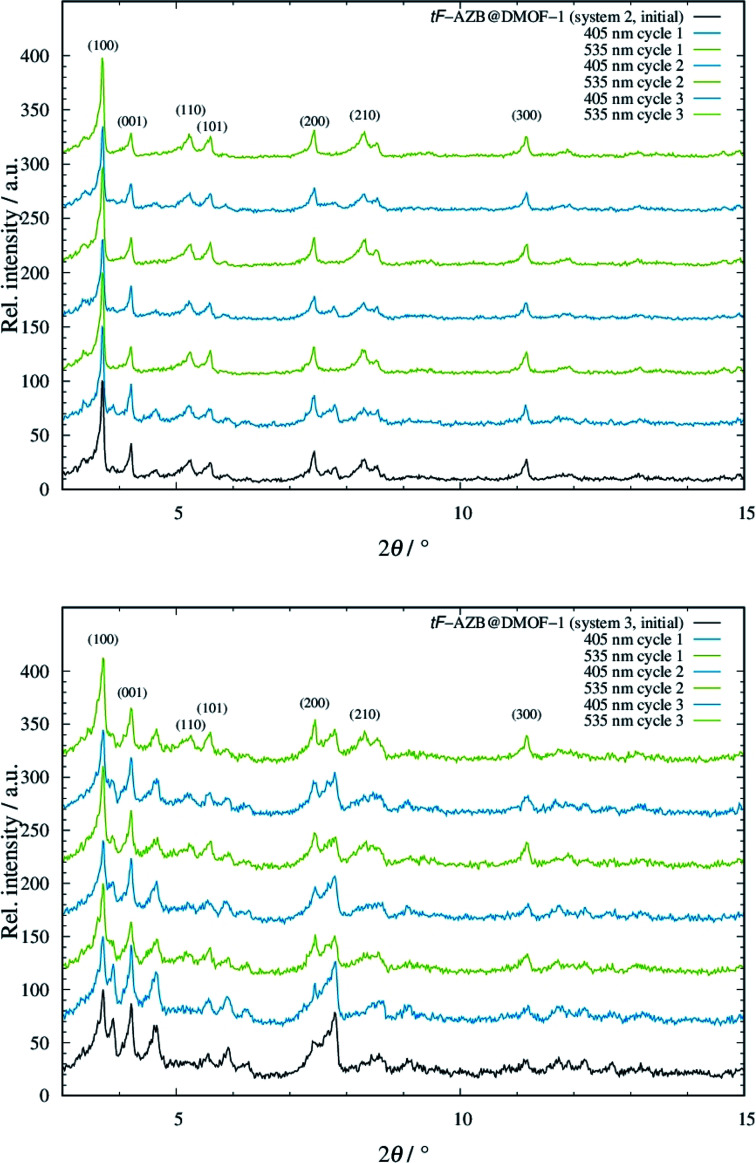
Diffraction patterns of system 2 (top) and system 3 (bottom), when non-irradiated (black line), irradiated with violet light (blue lines), and green light (green lines) with off-set. The diffraction patterns were measured at 298 K (Stoe Stadi P: *λ* = 0.7093 Å).

To take advantage of the light induced isomerization processes, we investigated potential structural guest-to-host transmission *via* XRPD measurements for systems 2 to 5. System 1 was not considered due to the low switching efficiency. In Fig. 5, the resulting diffraction patterns of systems 2 and 3 are depicted. The respective diffraction patterns of the systems 4 and 5 can be found in Fig. S22, top and bottom, ESI.†

For both systems 2 and 3, modulation of reflection intensities occurs as a function of irradiation. Upon violet light irradiation, reflections originating from the orthorhombic phase gain intensity, which is especially visible in the low angle region at approx. 3.9° and 4.6° 2*θ*. By green light exposure, these reflections loose intensity but those at *e.g.* approx. 8.3° 2*θ* get more intense. The pattern of the initial phase of both systems 2 and 3 shows characteristics of both the patterns after violet and green light exposure. This is not surprising, as from UV/Vis and NMR measurements a mixture of *E* and *Z* isomer was found to be present. Even though changes in the diffraction patterns were observed, all of these are small, which is in analogy to the work of Agarkar and Das,^37^ but in contrast to the reported results by Kitagawa and co-workers on non-substituted azobenzene.^42^ When going to even higher dilutions such as in systems 4 and 5, the *E*-to-*Z* conversion does not affect the host structure at all (see Fig. S22, ESI†). These findings are surprising due to the significantly higher switching efficiency of tF-AZB inside DMOF-1 compared to non-fluorinated azobenzene. Interactions between the azobenzene molecules and the DMOF-1 scaffold are assumed to be stronger than those between PAP or tF-AZB and the MOF host. Consequently, light induced isomerization of the first leads to structural changes of the host, whereas *E*-to-*Z* conversions of the latter cause almost no change. These assumption were further strengthened *via* IR spectroscopic measurements on a 1 : 1 (system 2) and a 0.125 : 1 (system 4) hybrid material. Here, the light induced *E*-to-*Z* conversion was found for the characteristic bands (see Fig. S23, ESI†). However, the IR bands of the host lattice remained unchanged, which points to weak interactions between tF-AZB and DMOF-1 (see Fig. S24, ESI†). Furthermore, molecular dynamics simulations were performed. Fig. S25, ESI,† displays a comparison of the IR spectra obtained from the MD simulations with the corresponding experimental data. Due to the rigid-body treatment of all X–H bonds, modes in the high frequency domain are not accessible. Nevertheless, in the range from 500 to 1800 cm^−1^ the calculated spectra are in good agreement with the experimental reference. Since molecular vibrations depend on the second derivative of the potential energy with respect to the nucleic degrees of freedom, they provide a sensible measure to assess the quality of the simulations in addition to the good agreement already observed for the structural properties. Additionally, simulations provide access to the microscopic properties of every atom in the system at a given time step, enabling a separate analysis of the vibrational properties associated to the ligand molecule, which are depicted in Fig. S26, ESI.†

### Determination of host–guest interactions inside tF-AZB_0.125_@DMOF-1

By applying quantum-chemical calculations, the obtained optical and structural properties of the obtained tF-AZB@DMOF-1 systems were studied in detail as a function of host–guest interactions inside a 2·2·2 supercell of DMOF-1. From the obtained UV/Vis- and NMR-spectroscopic data, the position of the *E* isomer is expected to be more stable than the position of the *Z* isomer, which causes a 100% *E* but only approx. 60% *Z* isomer being generated upon violet and green light exposure, respectively.

**Fig. 6:**
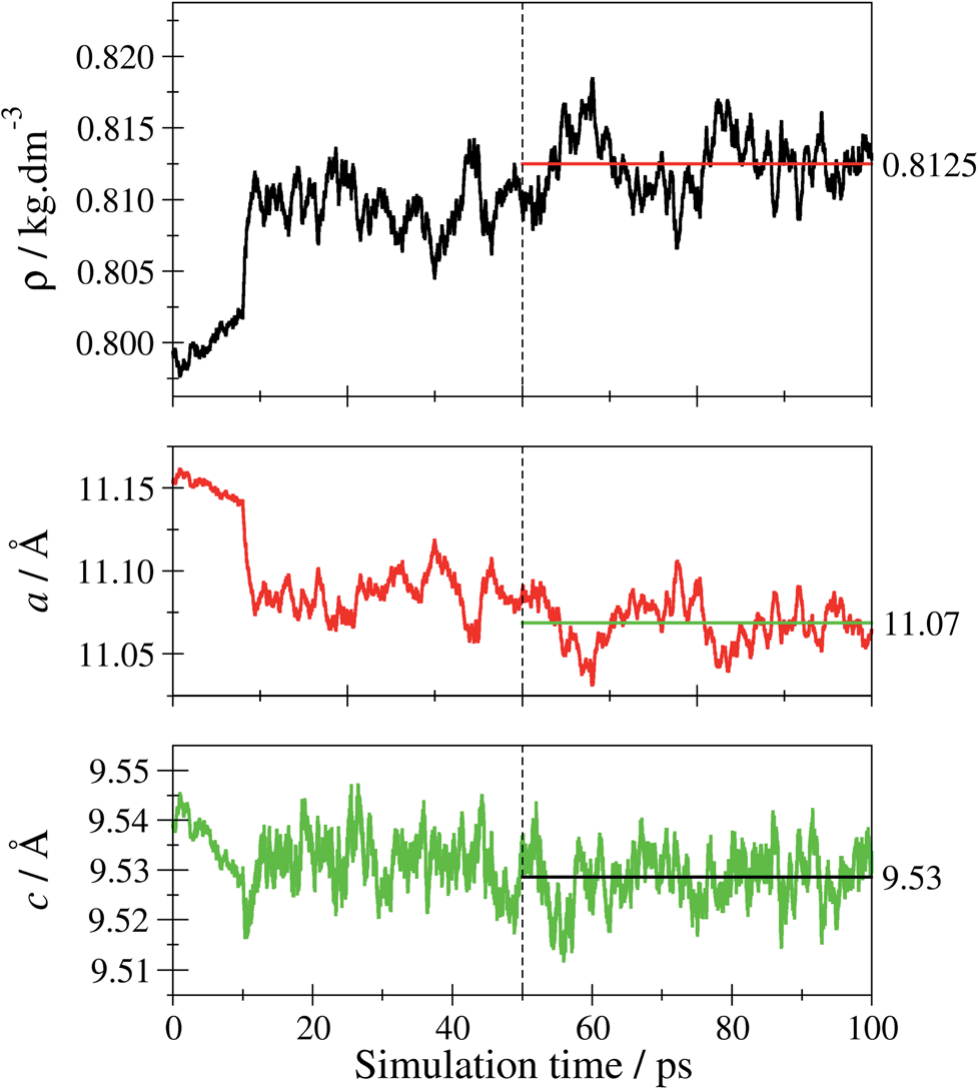
Time-evolution of the density *ρ* and the lattice parameters *a* and *c* of tetragonal DMOF-1 over the simulation period. Only the last 50 ps of the trajectory have been considered, when determining the respective average values shown as horizontal lines.

Prior to studying the tF-AZB@DMOF-1 guest–host complexes, the properties of the empty host lattice in absence of the guest molecule were evaluated to assess the performance of the SCC DFTB/3ob level of theory. Fig. 6 depicts the time evolution of the system's density and the lattice parameters over the simulation period.

The average density 〈*ρ*〉 evaluated over the second half of the sampling period is obtained as 0.8125 kg dm^−3^ corresponding to an average cell volume 〈*V*〉 of 2335 Å^3^. This value is in excellent agreement with the theoretical and experimental estimates for the equilibrium volume *V*_0_ of DMOF-1 reported as 2334 and 2301 Å^3^ (ref. 77) corresponding to densities of 0.8127 and 0.8224 kg dm^−3^, respectively. Similarly, the average lattice parameters 〈*a*〉 and 〈*c*〉 obtained from the simulation as 11.07 and 9.53 Å compare well to the experimental values of 10.93 und 9.61 Å obtained for DMOF-1 at 223 K.^50^ In order to further verify the structural properties, the associated X-ray powder diffraction (XRPD) pattern has been calculated. In addition to just computing the XRPD pattern for a 0 K structure by employing the respective minimum geometry (considering also the relaxation of the lattice parameters yielding *a*_min_ = 11.19 Å and *c*_min_ = 9.56 Å), the room-temperature XRPD pattern was determined by averaging the XRPD patterns obtained for every 5th configuration taken from the second half of the sampling period. The resulting average over 5000 individual XRPD patterns is shown in Fig. S27, ESI.† Comparison to the experimental reference pattern shows that the thermally averaged XRPD pattern is notably improved over the 0 K result (see Fig. S28, ESI†). This validation confirms the SCC-DFTB/MD framework being an adequate method to describe the DMOF-1 host lattice at elevated temperatures.

**Fig. 7:**
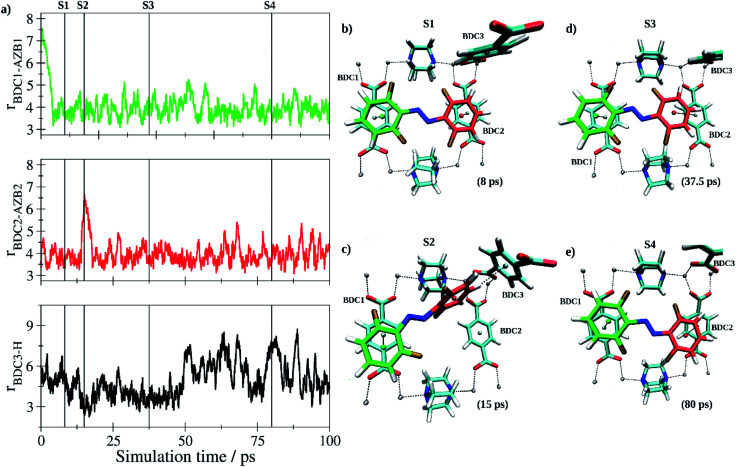
(a) Key distances between *E*-tF-AZB and BDC^2−^ groups of the DMOF-1 host scaffold based on the centroids of the aromatic units determined as average over the respective carbon atoms. In addition, the H-centroid distance between the aromatic moieties of *E*-tF-AZB highlighted in red and the BDC3 group of the host lattice is shown (bottom) indicating the formation of a short-lived configuration displaying a T-stacking interaction (see also snapshot S2). (b and e) Snapshots displaying representative configurations of the *E*-tF-AZB@DMOF-1 interaction taken from the simulation trajectory. The associated time steps are marked as S1 to S4 in the time series.

In the further process, two independent simulations of the *E*- and *Z*-tF-AZB isomers inside the DMOF-1 scaffold were carried out. Both the *E* and *Z* form have been pre-optimized and randomly inserted into one of the pores ensuring a minimum distance of 1.25 Å to all atoms of the host matrix. Following a re-equilibration period of 12.5 ps, data sampling was performed for 100 ps in each case. Each isomer quickly adopted a suitable interaction motif and remained in the respective binding site until the end of the simulation being only subject to thermal fluctuations. As expected, the *E* isomer of tF-AZB was binding to the aromatic moieties of two separate BDC^2−^ units. Fig. 7 depicts the time evolution of key distances between the two aromatic rings in *E*-tF-AZB and the relevant residues of the DMOF-1 host lattice based on the centroids of the aromatic rings determined as average over the respective carbon atoms.

At the beginning of the simulation, both aromatic rings of the guest molecule form the characteristic configuration associated with off-center parallel stacking.^78^ After a simulation time of 15 ps, the stacking between one of the difluoromethyl-rings of *E*-tF-AZB (highlighted in red in Fig. 7c) and the respective BDC^2−^ ring dissociated. Instead, this difluormethyl-group established a perpendicular T-stacking interaction with the aromatic ring of a third BDC^2−^ unit (see Fig. 7c). This configuration proofed to be a short-lived species, however, and the initial coordination was quickly re-established. This interaction motif persisted for the remainder of the simulation, but the interaction was not static. Due to thermal excitation, the tF-AZB molecule displayed translational and rovibrational oscillations about the binding site (see time series in Fig. 7a). However, these oscillations are not intense, pointing to a rather stable position of *E*-tF-AZB inside the DMOF-1. For low loading amounts, the calculated characteristics of the *E* isomer position inside DMOF-1 are in agreement with the obtained results by liquid-state NMR and UV/Vis spectroscopic measurements.

**Fig. 8:**
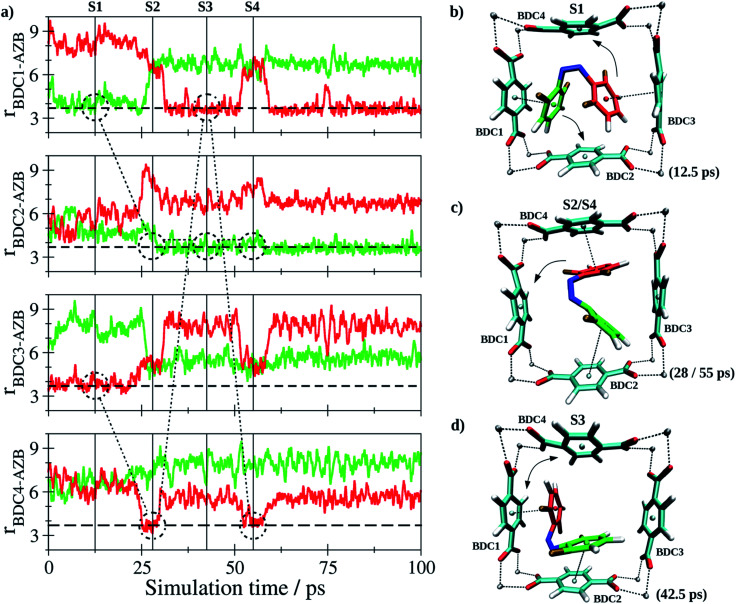
(a) Key distances between *Z*-tF-AZB and BDC^2−^ groups of the DMOF-1 host lattice based on the centroids of the aromatic units determined as average over the respective carbon atoms. The variation of the nearest BDC^2−^ groups resulting from the rotation of *Z*-tF-AZB in the lattice is indicated by the dashed and dash-dotted markers for the two aromatic moieties colored in red and green. (b and d) Snapshots displaying representative configurations of the *Z*-tF-AZB@DMOF-1 interaction taken from the simulation trajectory. The associated time steps are marked as S1 to S4 in the time series.

In case of *Z*-tF-AZB, an entirely different interaction motif is observed (see Fig. 8).

Since the *Z* form of the molecule is more compact, it cannot form interactions to two adjecant BDC^2−^ units at the same time. Instead, the interaction between two perpendicular BDC^2−^ residues was established (see screenshots in Fig. 8b–d). Again, the dynamics of this interaction are evaluated based on the centroids of all involved aromatic rings. Although the guest molecule remained in this binding site over the entire sampling phase, a notable degree of freedom in terms of rotational motion inside the binding site formed by the residues labeled BDC1-4 was observed. Consequently, the aromatic moieties of the guest-molecule were interacting with different BDC^2−^ units over the course of the simulation. The variation in the nearest BDC^2−^ groups is highlighted by the dashed and dash-dotted markers inserted in Fig. 8a. While oscillations for the *E* isomer were shown to occur in a small range, a high mobility of the *Z* isomer was found. Instead of one preferred binding site such as for the *E* form, the *Z* isomer exhibits a high degree of mobility inside the MOF pore. Again, this nicely corroborates the observations that only approx. 60% *Z* isomer can be generated upon green light exposure (see Table 3). Conclusively, the degree of potential binding sites is assumed to have a significant impact on the *E*/*Z* ratio. To exclude this being the result of differing host–guest interactions strengths, these were estimated by determining the respective interaction energy *U*_int_ for both isomers according to

1 *U*_int_ = *U*_tF-AZB@DMOF-1_ − 〈*U*_tF-AZB_〉 − 〈*U*_DMOF-1_〉

**Fig. 9:**
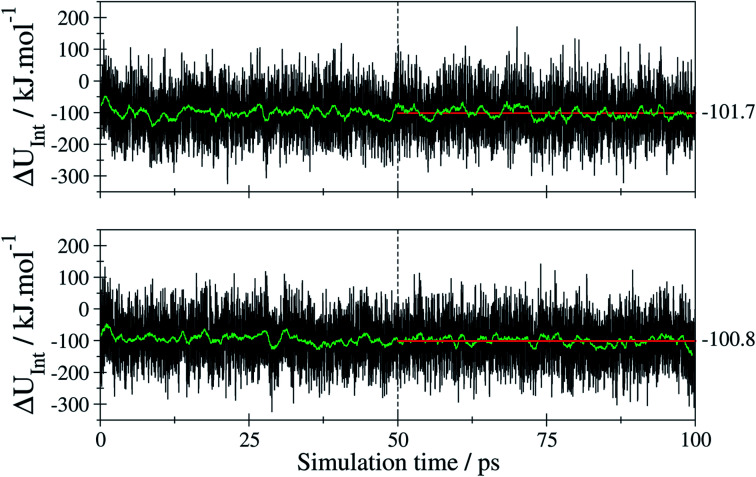
Time series of the interaction energy (black) and the associated running average over 250 data points (green) obtained for *E*-tF-AZB (top) and *Z*-tF-AZB (bottom) in DMOF-1 *via* the SCC DFTB MD simulations. The associated averages (red) have been evaluated employing only the last 50 ps of the trajectory.

With *U*_tF-AZB@DMOF-1_ being the total potential obtained for the *E*/*Z*-tF-AZB@DMOF-1 interaction at a given time step of the MD simulation, 〈*U*_tF-AZB_〉 and 〈*U*_DMOF-1_〉 represent the average potential energy obtained from separate MD simulations of isolated *E*- or *Z*-tF-AZB and DMOF-1 at the same conditions. The respective time series of *U*_int_ obtained for both isomers are depicted in Fig. 9.

The average values of −101.7 and 100.8 kJ mol^−1^ for the *E* and *Z* isomer have been determined considering only the last 50 ps of the simulation trajectory. These values indicate that despite the differences in the configuration both isomers show a similar interaction when binding to the host framework. This means that despite the different amounts of binding sites, both isomers interact equally strongly with the host framework. Therefore, the efficiency of *E*-to-*Z* conversion and *vice versa* is exclusively the result of isomer mobility.

## Conclusion

In this study, the photochromic behavior of tF-AZB inside DMOF-1 was investigated as a function of host–guest and guest–guest interactions. tF-AZB was chosen as photochromic guest molecule due to its visible light response. That way, UV light was no longer required to initiate the light induced isomerization processes. Different tF-AZB : DMOF-1 molar ratios were applied in order to decrease possible guest–guest and enhance host–guest interactions. In total, five photoactive systems with varying optical properties were obtained. At a maximum loading of 1.5 tF-AZB per formula unit DMOF-1, system 1 displays the least efficient photoactive system: Upon violet light irradiation, 93% *E* isomer was generated, but green light irradiation resulted in only ∼40% *Z* isomer. These low switching yields probably originate from the dense packing inside the MOF pores. Diminishing the loading degree to a 1 : 1 and even more to a 0.125 : 1 tF-AZB : DMOF-1 molar ratio led to almost quantitative switching between the *E* and *Z* isomer in systems 2 to 5 (100% *E* and 60% *Z*, respectively). The isomerization processes were proved to be reversible over ten switching cycles. Surprisingly, this successful *E*-to-*Z* conversions do not significantly affect the structure of the host. In order to understand the origin of the non-responsive structural behavior, molecular dynamics simulation on one tF-AZB inside a 2·2·2 DMOF-1 supercell, which corresponds to the composition in systems 4 and 5, was applied. By this, the preferred interaction sites of the two isomers could be characterized in detail. In case of the *E* isomer, the expected off-center parallel stacking interaction to two adjacent BDC^2−^ residues was observed. In addition, a short-lived configuration displaying T-stacking could be identified as well. Due to its compactness, the *Z* isomer showed an entirely different interaction motif. While still binding two BDC^2−^ units of the host scaffold, these residues are in a perpendicular arrangement. In addition, *Z*-tF-AZB displayed an increased mobility in its binding site, showing a re-arrangement of the binding *via* rotation in the pore of the host lattice. Irrespective of these entirely different modes of interaction, both isomers display a similar binding energy to the host framework and it can be concluded, that the difference in the *E*/*Z* conversion rate found in the experiments is due to respective isomer mobilities. In this work, the molecular dynamics simulations were limited to study just a single tF-AZB molecule inside DMOF-1. While simulations of higher loading ratios are in principle possible as well, the effects due to crowding inside the pores of the MOF host are expected to result in a low diffusivity of the individual molecules that may be beyond the time scales accessible in SCC DFTB MD simulations. Therefore, our current research focuses on developing experimental methods to follow the positional changes for a single molecule, but also for higher loading degrees. Common diffraction (XRD) reaches its limits here, since the high mobility of the guest molecules cannot be resolved by long-range order techniques. Instead, local probes *via* solid-state NMR (ssNMR) and total scattering coupled to PDF will allow for a detailed analysis of such partially amorphous materials. The power of the combination of these techniques has been impressively shown for sodium nitroprussides inside silica monoliths,^79^ biocompatible amorphous matrices,^80^ and an amorphous SiO_2_ matrix.^81^ Furthermore, the structure of water inside bioactive glasses was studied *via* total X-ray scattering techniques.^82,83^ Tracing the confinement of photoswitchable dyes inside MOFs will pave the way to an unprecedented insight of the hybrid system's structure and its related optical properties.

## Experimental section

Commercially available 1,4-diazabicyclo[2.2.2]octane (Sigma Aldrich), 2,6-difluoroaniline (Alfa Aesar), *N*,*N*′-dimethylformamide (n.i.), chloroform (ZEUS), lead(iv)-acetate (Alfa Aesar), terephthalic acid (Alpha Aesar), and [Zn(NO_3_)_2_] (n.s.) were used without any further purification.

### *Ortho*-tetrafluoroazobenzene synthesis

2,6-Difluoro aniline (353.0 mg, 2.73 mmol) was dissolved in chloroform (25 ml) and lead(iv)-acetate (3.03 g, 6.83 mmol) was added. The colour of the suspension changed from colourless to orange. The suspension was heated for 1.5 hours under reflux (70 °C) and afterwards stirred overnight at room temperature. The crude residue was filtered, and the solution was washed three times with glacial acetic acid (50%). The organic phase was dried out over sodium sulphate. The solvent was removed under reduced pressure. The residue was purified using column chromatography (dichloromethane/cyclohexane 3 : 2) and obtained as an orange salt. The purity of *ortho*-tetrafluoroazobenzene was confirmed by ^1^H- and ^19^F-NMR spectroscopy (see Fig. S29 and S30, ESI†).

^1^H-NMR (300 MHz, DMSO-d_6_): *δ*/ppm = 7.68–7.58 (m, 2H, *E*-4H, 4′H), 7.49–7.34 (m, 2H, *Z*-4H, 4′H and m, 4H, *E*-3H, 3′H, 7H, 7′H), 6.21–6. 51 (m, 4H, *Z*-3H, 3′H, 7H, 7′H).

^19^F-NMR (282 MHz, DMSO-d_6_): *δ*/ppm = −120.99 (m, 4F, *Z*-2F, 2′F, 6F, 6′F), −121.9 (m, 4F, *E*-2F, 2′F, 6F, 6′F).

### DMOF-1 synthesis

Zn(NO_3_)_2_·6H_2_O (125.0 mg, 0.42 mmol), terephthalic acid (70.0 mg, 0.42 mmol) and dabco(1,4-diazabicyclo[2.2.2]octane) (20.0 mg, 21.0 mmol) were mixed with DMF (dimethylformamide) (3 ml) in a 8 ml Teflon lined autoclave. The mixture was heated (120 °C, 2 days) in an oven and cooled down to room temperature afterwards. The resulting colourless powder was filtered, then washed with a small amount of DMF and dried on air overnight. To remove embedded DMF molecules, the residue was heated under reduced pressure (120 °C, 24 h) and stored under argon atmosphere. The phase purity of DMOF-1 was checked by XRPD (see Fig. S31, ESI†).

### Preparation of tF_*x*_-AZB@DMOF-1 systems

DMOF-1 and tF-AZB in the molar ratios 3 : 1, 1 : 1 and 0.125 : 1 (tF-AZB : DMOF-1) were mixed under an argon atmosphere. The resulting homogenous powder was placed into a small glass vessel inside a Schlenk tube and heated at elevated temperatures under reduced pressure ∼9.4 × 10^−2^ mbar for several hours in the dark. For system 1, a maximum temperature of 70 °C was chosen, whereas systems 2 and 4 were heated to 65 °C and system 3 and 5 to 105 °C. The excess of tF-AZB resublimed at the top of the glass tube. To prevent the absorption of water and decomposition upon contact with air and moisture, all compounds were stored in a glovebox under argon atmosphere. Furthermore, the sample was kept in the dark to avoid any undesired switching process.

The phase purity of the resulting materials was checked by XRPD measurements. The XRPD patterns of the dilutions are shown in Fig. S1 to S5.† The exact composition was obtained from liquid-state NMR-spectroscopic measurements. Details are given in Fig. S7–S11, ESI.†

### X-ray powder diffraction

To check the purity of the crystalline samples, laboratory measurements were carried out on a Stoe Stadi P diffractometer (Stoe, Darmstadt, Germany) in transmission geometry with Mo-K_α1_-radiation (*λ* = 70.93 pm) utilizing a focusing Ge(111) primary beam monochromator and a Mythen 2 DCS4 detector. The measurement was performed in the 2*θ* range of 2.0°–40.4° with a step size of 0.015°. The respective powder was sealed in a glass capillary under argon atmosphere to prevent absorption of humidity. In order to follow light-induced structural guest-to-host transmission, XRPD patterns were collected after 15 min of 405 nm and 535 nm irradiation, respectively. For this purpose, the capillary was rotated further on the goniometer head, so that the light exposure was as even as possible. Subsequently, the diffraction pattern was collected. All these steps were performed in the dark to prevent any daylight to reach the sample. For the illumination of the hybrid materials, a Prizmatix PRI FC5-LED-WL (five high power Fiberglas coupled LEDs output with potentiometer for manual power control) was used.

### Reflection spectroscopy

Reflection spectra of the tF-AZB@DMOF-1 systems were recorded using an Agilent Cary 5000 UV-Vis-NIR Spectrophotometer. Therefore, the powder was placed into a sample holder under argon atmosphere to prevent absorption of humidity. Spectra were recorded in the range of 200 nm to 700 nm before and after irradiation (*λ* = 405 nm or *λ* = 535 nm, 5 min). Detailed information on the irradiation processes are given in the respective spectra. For the illumination of the hybrid materials, a Prizmatix PRI FC5-LED-WL (five high power Fiberglas coupled LEDs output with potentiometer for manual power control) was used.

### Liquid-state NMR spectroscopy

^1^H-NMR spectra were collected on a 300 MHz Bruker Avance DPX NMR spectrometer equipped with a 5 mm broadband probe. The solvent served as an internal reference (*δ*_H_ (CDCl_3_) = 7.24 ppm, *δ*_H_ (DMSO-d_6_) = 2.50 ppm).^19^F spectra were recorded on a 400 MHz Bruker Avance 4 Neo spectrometer. All measurements were carried out at room temperature and processed with MestReNova 9.0.1-13254.

For liquid-state NMR measurements on tF-AZB, small amounts of tF-AZB were dissolved in DMSO-d_6_. For liquid-state NMR measurements on the hybrid systems, approx. 3.0 mg of tF_*x*_-AZB@DMOF-1 were digested in 0.5 ml of DMSO-d_6_ and 25 μl of DCl were added. Directly after the addition of DCl, the sample was placed in an NMR glass tube. The spectra were recorded *ca.* 2 min after treatment with DCl. In order to determine the composition and the *E*/*Z* ratios before and after irradiation, characteristic proton signals were integrated and related to each other. The respective NMR spectra can be found in the ESI (Fig. S7 to S21†). Furthermore, NMR spectra of the irradiated samples (violet light and green light exposure, respectively) were collected. For this purpose, the samples were irradiated for 15 min. For the illumination of the hybrid materials, a Prizmatix PRI FC5-LED-WL (five high power Fiberglas coupled LEDs output with potentiometer for manual power control) was used.

### Infrared spectroscopy

In order to understand the occurring host–guest and guest–guest interactions, systems 2 to 5 and pure DMOF-1 were analyzed *via* IR spectroscopy. The measurements were carried out on a BRUKER Alpha II FT-IR-Spectrometer under argon atmosphere to prevent absorption of humidity. The hybrid materials were prepared as explained in the following: a half of a spatula tip of the sample was thoroughly ground with two spatulas of KBr. Afterwards, the mixture was pressed for 30 min at a pressure apparatus with a set pressure of ∼2 tons yielding a thin and transparent light-colored pellet. The pellet was then placed into the IR spectrometer sample holder. Scans were done in the range of 360 cm^−1^ to 4000 cm^−1^ with a resolution of 2 cm^−1^ and 90 scans per sample. The background was determined by preparing a pure KBr pellet and measuring it with the same instrument settings as the sample. Furthermore, IR spectra of the irradiated samples (violet and green light exposure, respectively) were collected. For this purpose, the samples were irradiated for 5 min. All measurements were carried out at RT and evaluated with the program OPUS version 8.2 build 8, 2, 28 (20190310) Copyright© Bruker Optic GmbH. For the illumination of the hybrid materials, a Prizmatix PRI FC5-LED-WL (five high power Fiberglas coupled LEDs output with potentiometer for manual power control) was used. The spectra can be found in Fig. S23 and S24, ESI.†

### Molecular dynamics simulation

The starting structure of DMOF-1 has been generated based on available crystal structure data^50^ extending the system to achieve a tetragonal 2·2·2 supercell. All SCC DFTB calculations have been carried under periodic boundary conditions using the DFTB+ code^84^ employing the 3ob parameter set.^85–87^ The integration along the axes of the Brillouin zone was performed using Monkhorst–Pack sampling^88^ based on a (2·2·2) extension of the lattice. All interactions between non-hydrogen and hydrogen atoms (*i.e.* X–H) have been subject to damping using a damping factor *ζ*_XH_ of 4.0 as required by the 3ob parameterization.^85^ To properly take the influence of dispersion contributions into account, the Grimme D3 correction^89^ was applied. The SCC-DFTB iterations were performed using a convergence criterion in the SCC error of 10^−4^ ensuring a convergence in energy of 10^−6^ Hartree.

The employed 3d-periodic DFTB/MM framework was implemented in our previously developed QM/MM MD simulation program^60–62^ interfaced to the DFTB+ package. Each atom in the cell was considered as irreducible as required by the molecular dynamics framework. The integration of the equations of motion was performed using the velocity Verlet algorithm.^90,91^ In order to achieve an MD time step of 2.0 fs all bonds, involving hydrogen atoms have been constraint using the Shake/Rattle algorithms.^92,93^ The Nose–Hoover chain thermostat^94^ (chain-length of 5) and the Berendsen manostat^95^ (relaxation time 10 ps) have been applied to achieve an isothermal-isobaric (*NPT*) ensemble along the simulation. The respective target temperature and pressure were set to 298.15 K and 1 atm, respectively. To account for the tetragonal nature of the MOF-lattice, a semi-isotropic pressurization was considered by decoupling the adjustment of the simulation cell along the *a*- and *b*-directions from that along the *c*-axis of the system. Visualization of the trajectories has been performed using VMD.^96^

In order to assess the performance of the SCC DFTB/3ob method for the description of the system, DMOF-1 has been investigated in the absence of a guest molecule. Following extensive thermalization and pressurization periods of 25 000 and 50 000 MD steps (50 and 100 ps), data sampling was performed for a total of 50 000 MD steps resulting in a trajectory length of 100 ps. The associated X-ray powder diffraction (XRPD) patterns have been calculated using the command line interface to RIETAN-FP^97^ provided by VESTA.^98^ In addition to calculating the XRPD pattern for the minimum geometry of DMOF-1, the influence of thermal effects have been taken into account by averaging the XRPD patterns obtained for every 5th configuration in the sampling phase of the room-temperature MD simulation. The simulations containing a single *E*- or *Z*-tF-AZB guest molecule have been started from the same equilibrated DMOF-1 structure. In both cases, a pre-equilibrated tF-AZB molecule was inserted at a random position into one of the pores of the host structure. After re-equilibration for 12.5 ps sampling was performed for 50 000 MD steps for each conformer.

In order to assess the interaction energy between the guest molecule and the host lattice simulations of isolated *E*- and *Z*-tF-AZB had to be executed as well employing the same simulation setup, however without pressure coupling. Again, simulation times of 12.5 and 100 ps were set for the equilibration and sampling phase.

Infrared spectra have been determined *via* the Fourier transform of the electric charge flux correlation function^99,100^ employing the time-dependent velocity and charge data obtained from SCC-DFTB MD simulations. The correlation window used in the analysis was 250 frames at a spacing of 4 fs corresponding to a total correlation time of 1 ps. An exponential window function with a decay parameter of 2.5 ps^−1^ has been applied in the subsequent Fourier transform step.

## Conflicts of interest

There are no conflicts to declare.

## Supplementary Material

RA-011-D0RA10500G-s001
