# Supplementary material for: Structural, dynamical, and photochemical properties of ortho-tetrafluoroazobenzene inside a flexible MOF under visible light irradiation
Source: RSC Adv. 2021 Jan 22;11(7):3917–30. doi: 10.1039/d0ra10500g (PMC8694203; doi:10.1039/d0ra10500g)
Supplement: RA-011-D0RA10500G-s001 [file RA-011-D0RA10500G-s001.pdf]

Electronic Supplementary Material (ESI) for RSC Advances.
This journal is © The Royal Society of Chemistry 2021

Supporting Information

Markus Rödl[a], Samuel Kerschbaumer[a], Holger Kopacka[a], Laura Blaser[a], Felix R. S. Purtscher[a], Hubert Huppertz[a], Thomas S. Hofer[a,*] and Heidi A. Schwartz[a,*]

[a]*Institute of General, Inorganic and Theoretical Chemistry, University of Innsbruck, Innrain 80-82, A-6020 Innsbruck, Austria.*

Corresponding author main: Heidi A. Schwartz

E-mail: heidi.schwartz@uibk.ac.at

Corresponding author theory: Thomas S. Hofer

E-mail: t.hofer@uibk.ac.at

Content

Figures S1 to S5. XRPD patterns of systems **1** to **5** compared to unloaded DMOF-1 and *tF*-AZB.

Figure S6. Diffraction patterns of systems **1** to **5** compared to theoretical patterns of un-loaded DMOF-1 and benzene-loaded DMOF-1.

Figure S7 to S21. ^{1}H NMR spectra of systems **1** to **5** digested in 1 ml of DMSO-d_6 and 25 µl of DCl before and after irradiation with blue and green light, respectively.

Figure S22. Diffraction patterns of system **4** and system **5**, when non-irradiated, irradiated with blue light and green light.

Figures S23 to S24. IR spectra of systems **2** (top) and system **4** (bottom), when irradiated with blue light (blue lines) and green light (green lines) compared to pure DMOF-1.

Figure S25. Comparison of a) the calculated IR spectra obtained from the SCC DFTB MD simulations against b) the experimental reference for DMOF-1 as well as the *E*/*Z*-*tF*-AZB@DMOF-1 complexes. Bands in the high energy range are not accessible in this case due to the rigid-body constraints applied to all X-H bonds.

Figure S26. a) Calculated IR spectra determined from the SCC DFTB MD simulations for DMOF-1 as well as the *E*/*Z*-*tF*-AZB@DMOF-1 complexes. b) Calculated IR spectra obtained by considering only the contributions of the E/Z-tf-AZB molecules in DMOF-1.

Figure S27. Average XRPD pattern of DMOF-1 obtained by averaging 5000 individual patterns computed for every 5th configuration of the MD sampling phase.

Figure S28. Comparison of the XRPD pattern for empty DMOF-1 obtained via a single minimum configuration and thermal averaging over the MD trajectory against the experimental reference.

Figures S29 and S30. ^{1}H and ^{19}F NMR spectra of *tF*-AZB.

Figure S31. Diffraction pattern of unloaded DMOF-1 in comparison to the peak intensities and position calculated from theoretical data.

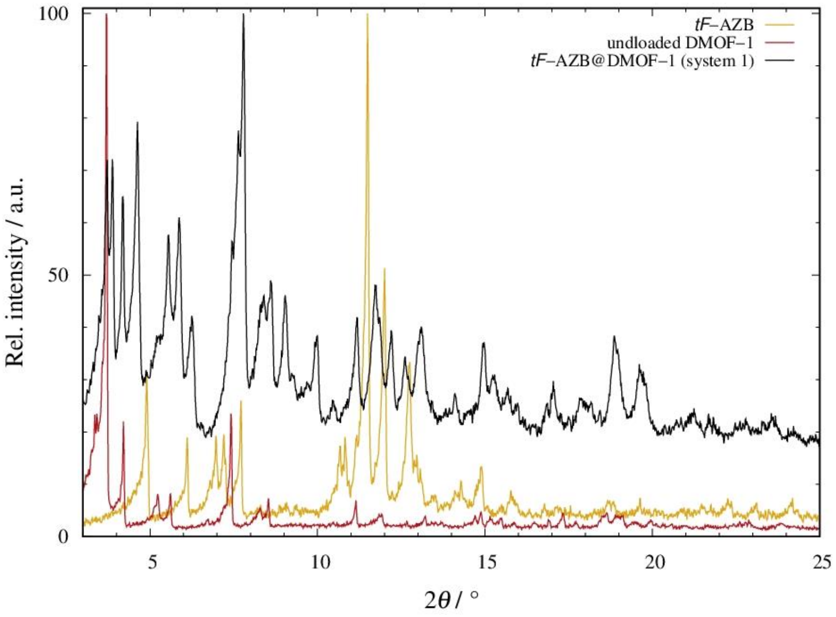

Figure S1. Diffraction pattern of unloaded *tF*-AZB@DMOF-1 (system **1**) (black line), pristine *tF*-AZB (orange line) and unloaded DMOF-1 (red line). The diffraction patterns were measured at 298 K (*Stoe Stadi P*: λ = 0.7093 Å).

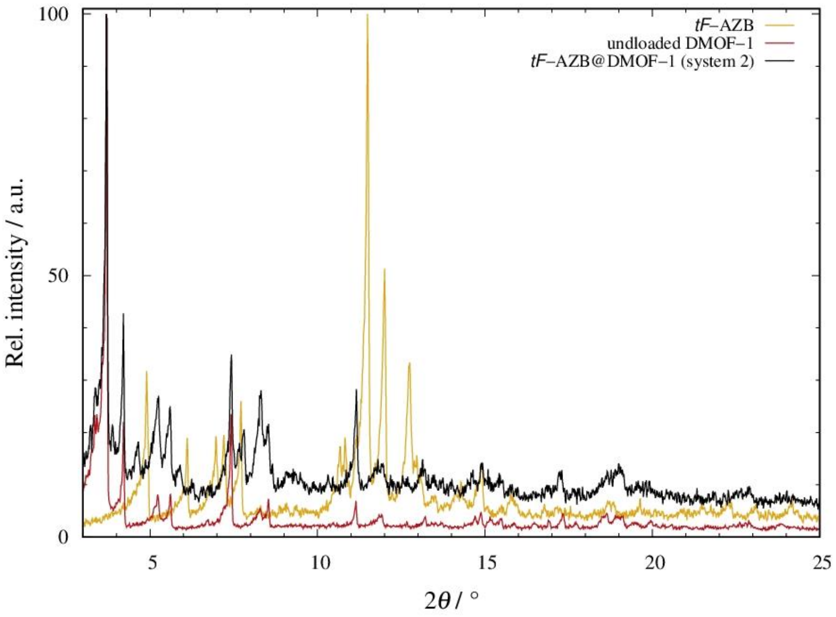

Figure S2. Diffraction pattern of unloaded *tF*-AZB@DMOF-1 (system **2**) (black line), pristine *tF*-AZB (orange line) and unloaded DMOF-1 (red line). The diffraction patterns were measured at 298 K (*Stoe Stadi P*: λ = 0.7093 Å).

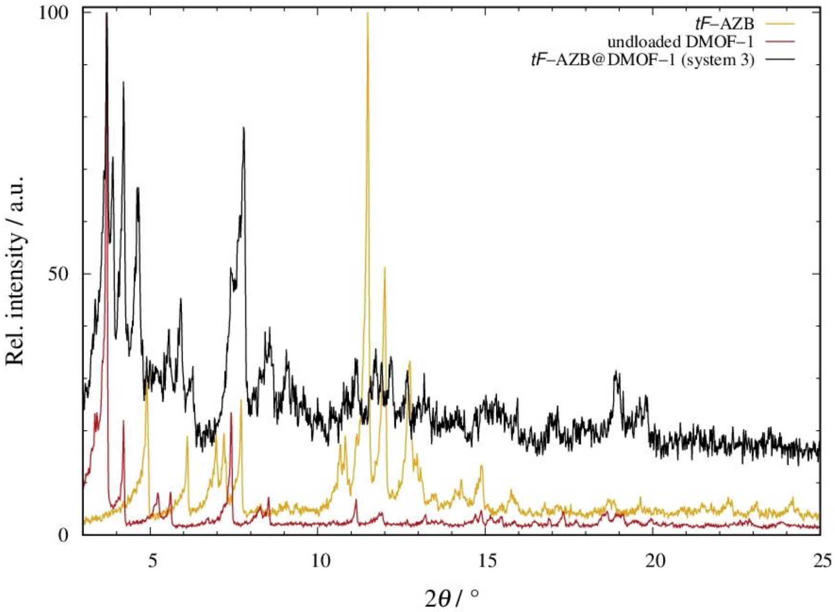

Figure S3. Diffraction pattern of unloaded *tF*-AZB@DMOF-1 (system **3**) (black line), pristine *tF*-AZB (orange line) and unloaded DMOF-1 (red line). The diffraction patterns were measured at 298 K (*Stoe Stadi P*: λ = 0.7093 Å).

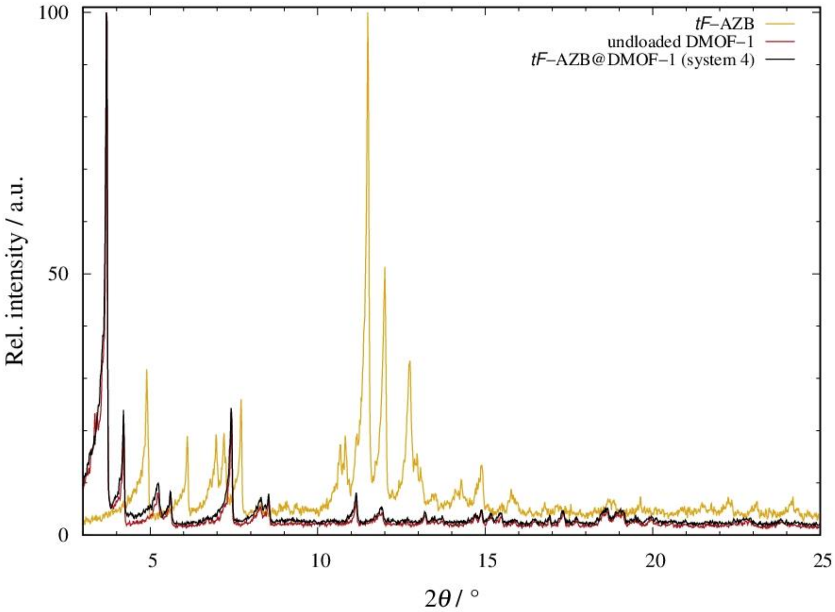

Figure S4. Diffraction pattern of unloaded *tF*-AZB@DMOF-1 (system **4**) (black line), pristine *tF*-AZB (orange line) and unloaded DMOF-1 (red line). The diffraction patterns were measured at 298 K (*Stoe Stadi P*: λ = 0.7093 Å).

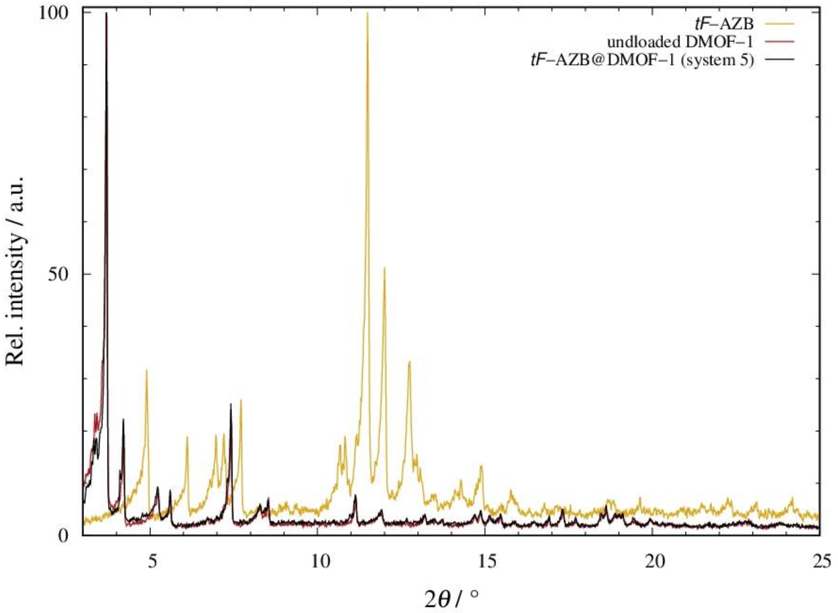

Figure S5. Diffraction pattern of unloaded *tF*-AZB@DMOF-1 (system **5**) (black line), pristine *tF*-AZB (orange line) and unloaded DMOF-1 (red line). The diffraction patterns were measured at 298 K (*Stoe Stadi P*: λ = 0.7093 Å).

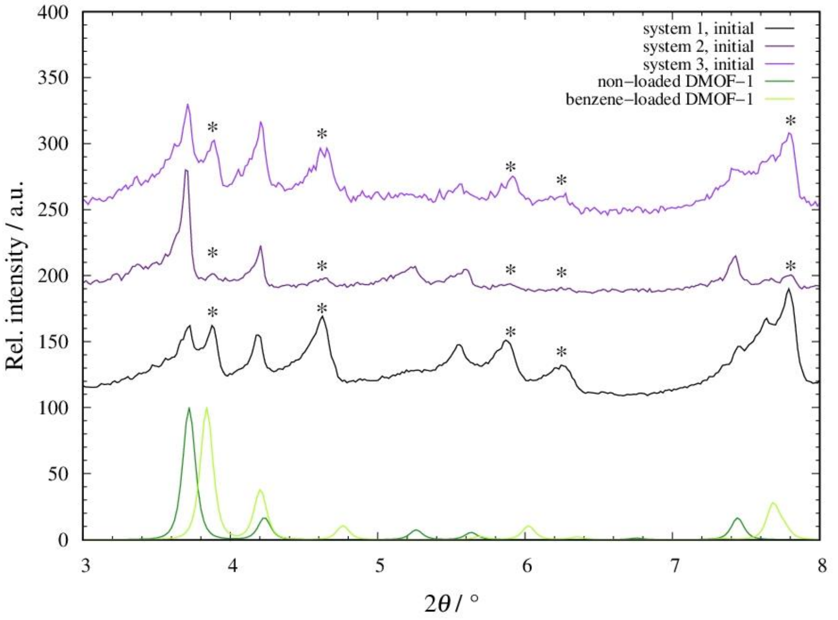

Figure S6. Diffraction patterns of systems **1** to **3** (purple lines) and theoretical patterns of un-loaded DMOF-1 (dark-green line), and benzene-loaded DMOF-1 (light-green line). The diffraction patterns of systems **1** to **5** were measured at 298 K (*Stoe Stadi P*: λ = 0.7093 Å).

NMR-spectroscopic investigations

Processing of the NMR data was conducted using *MestReNova 9.0.1-13254*. Here, the ratio of *tF*-AZB to DMOF-1 was calculated via integrating the respective proton signal of terephthalic acid (approx. 8 ppm) to the *Z* isomer and *E* isomer proton signals of *tF*-AZB following the work of *Benedict* and co-workers[2].

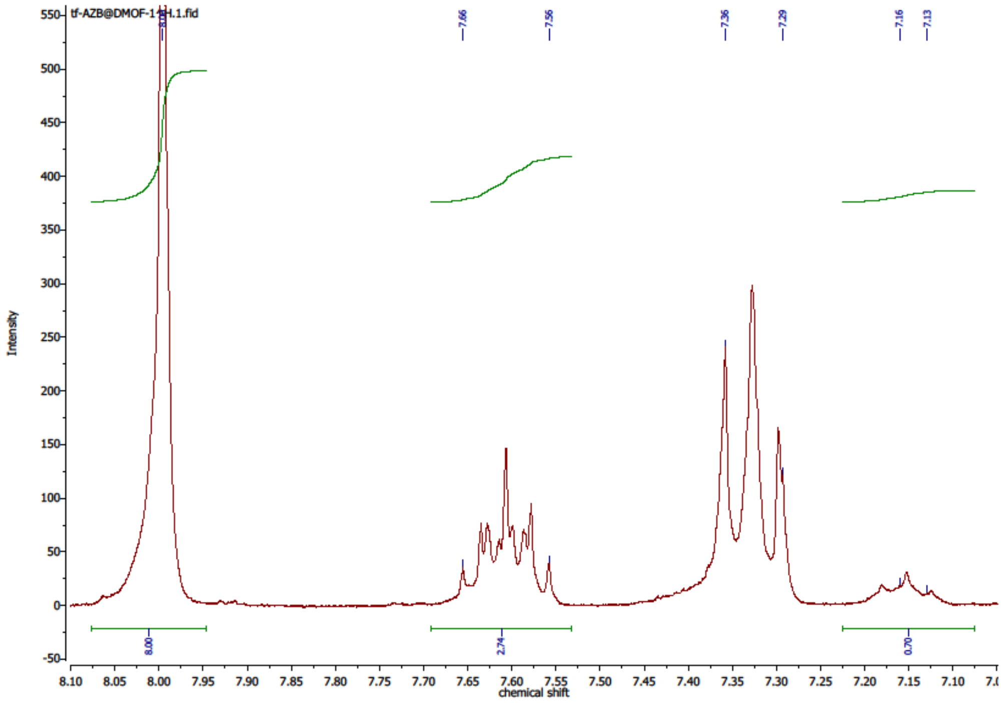

Figure S7. ^{1}H NMR spectrum of *tF*-AZB@DMOF-1 (system **1**) digested in 1 ml of DMSO-d_6 and 25 µl of DCl. Integration of the terephthalic acid protons and the *tF*-AZB protons indicate a ratio of 1.5:1 *tF*-AZB to DMOF-1. The *E*/*Z* ratio of the non-irradiated state was found to be 88.7:11.3.

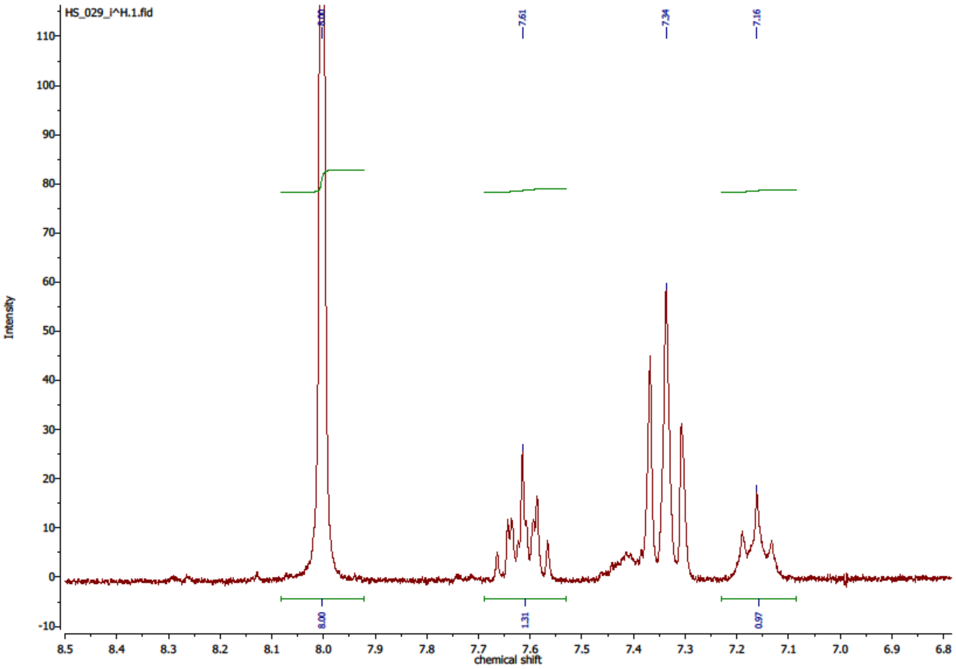

Figure S8. ^{1}H NMR spectrum of *tF*-AZB@DMOF-1 (system **2**) digested in 1 ml of DMSO-d_6 and 25 µl of DCl. Integration of the terephthalic acid protons and the *tF*-AZB protons indicate a ratio of 1:1 *tF*-AZB to DMOF-1. The *E*/*Z* ratio of the non-irradiated state was found to be 72.4:27.6.

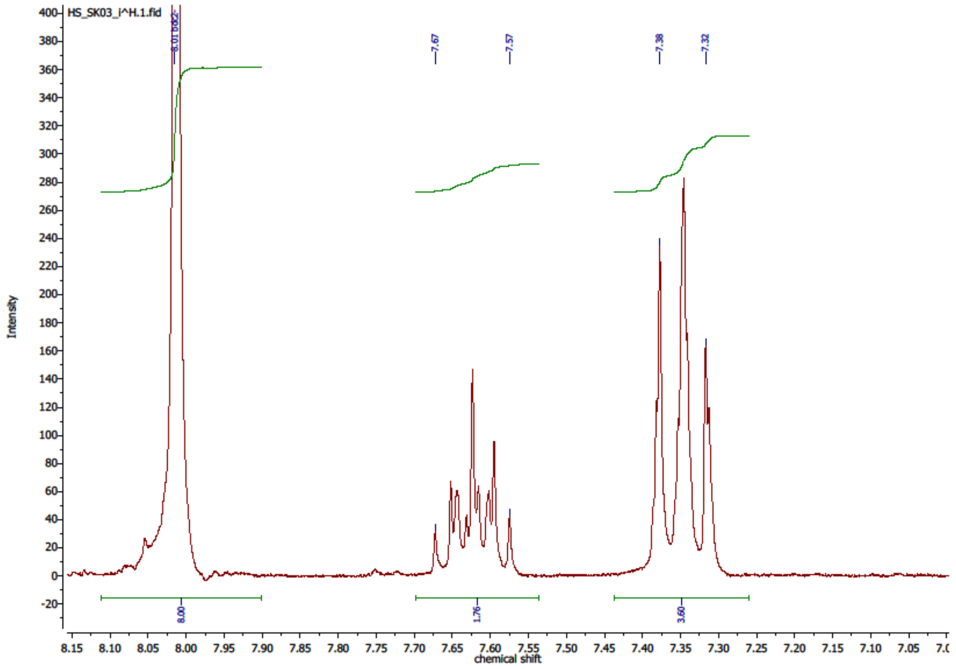

Figure S9. ^{1}H NMR spectrum of *tF*-AZB@DMOF-1 (system **3**) digested in 1 ml of DMSO-d_6 and 25 µl of DCl. Integration of the terephthalic acid protons and the *tF*-AZB protons indicate a ratio of 0.9:1 *tF*-AZB to DMOF-1. The *E*/*Z* ratio of the non-irradiated state was found to be 100% *E* isomer.

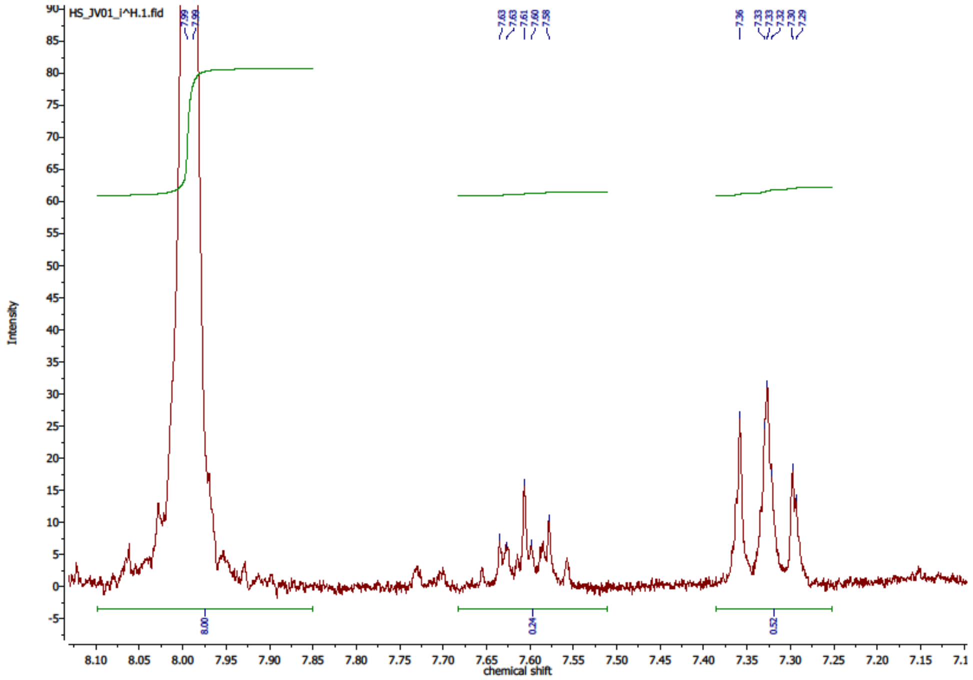

Figure S10. ^{1}H NMR spectrum of *tF*-AZB@DMOF-1 (system **4**) digested in 1 ml of DMSO-d_6 and 25 µl of DCl. Integration of the terephthalic acid protons and the *tF*-AZB protons indicate a ratio of 0.125:1 *tF*-AZB to DMOF-1. The *E*/*Z* ratio of the non-irradiated state was found to be 100% *E* isomer.

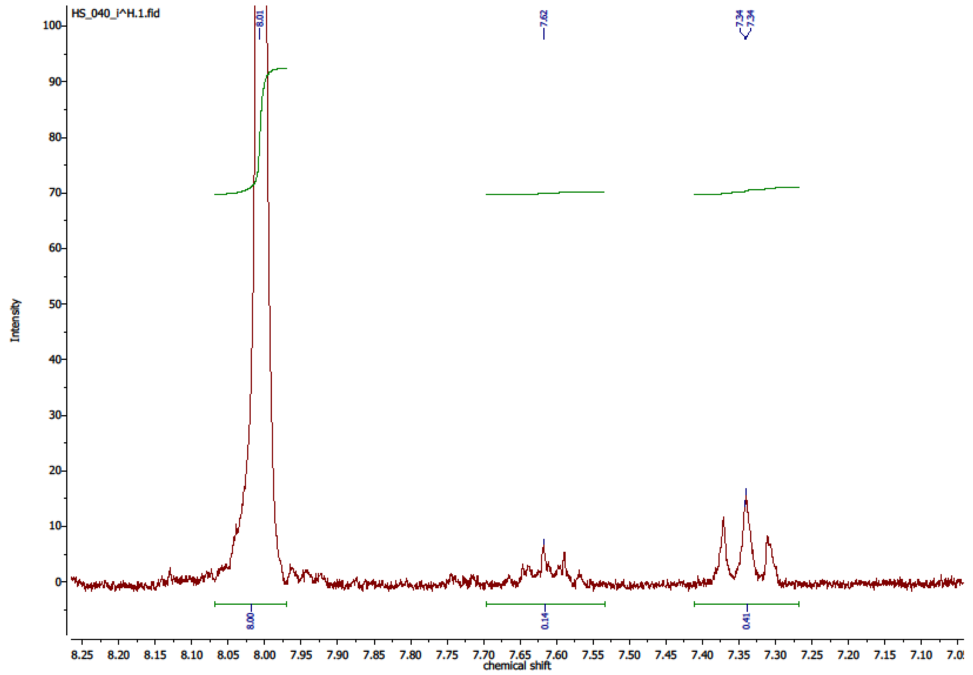

Figure S11. ^{1}H NMR spectrum of *tF*-AZB@DMOF-1 (system **5**) digested in 1 ml of DMSO-d_6 and 25 µl of DCl. Integration of the terephthalic acid protons and the *tF*-AZB protons indicate a ratio of 0.125:1 *tF*-AZB to DMOF-1. The *E*/*Z* ratio of the non-irradiated state was found to be 100% *E* isomer.

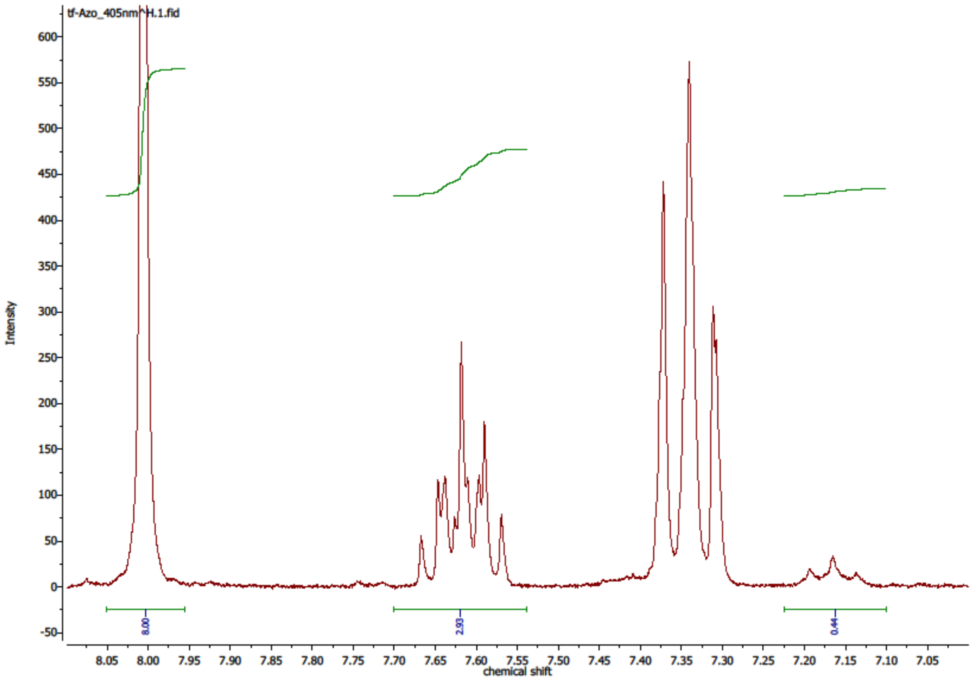

Figure S12. ^{1}H NMR spectrum of *tF*-AZB@DMOF-1 (system **1**) digested in 1 ml of DMSO-d_6 and 25 µl of DCl after irradiation with blue light (λ = 405 nm) for 15 min. The *E*/*Z* ratio was found to be 93% *E* isomer.

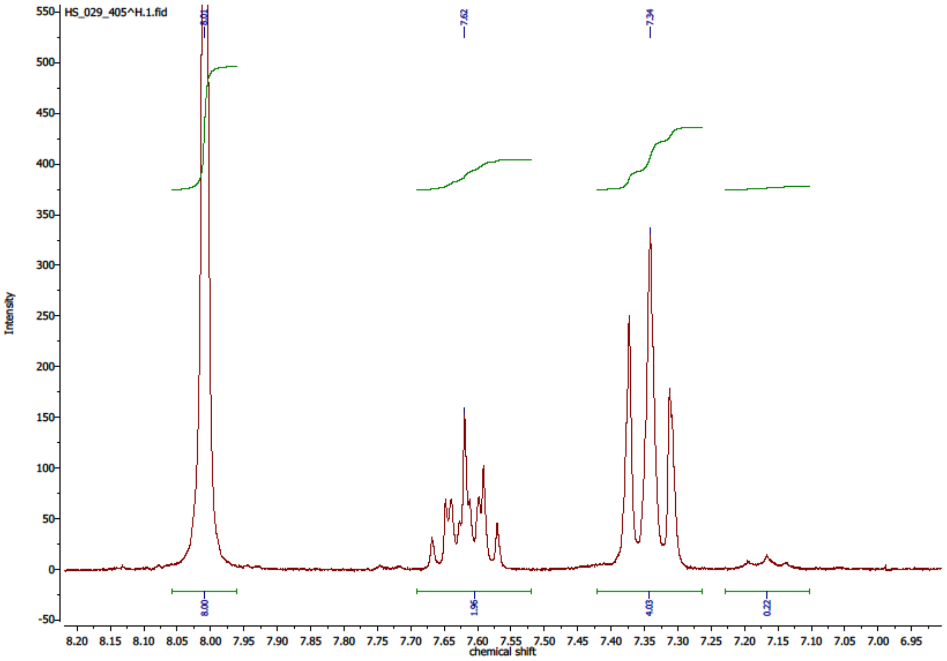

Figure S13. ^{1}H NMR spectrum of *tF*-AZB@DMOF-1 (system **2**) digested in 1 ml of DMSO-d_6 and 25 µl of DCl after irradiation with blue light (λ = 405 nm) for 15 min. The *E*/*Z* ratio was found to be 94.6% *E* isomer.

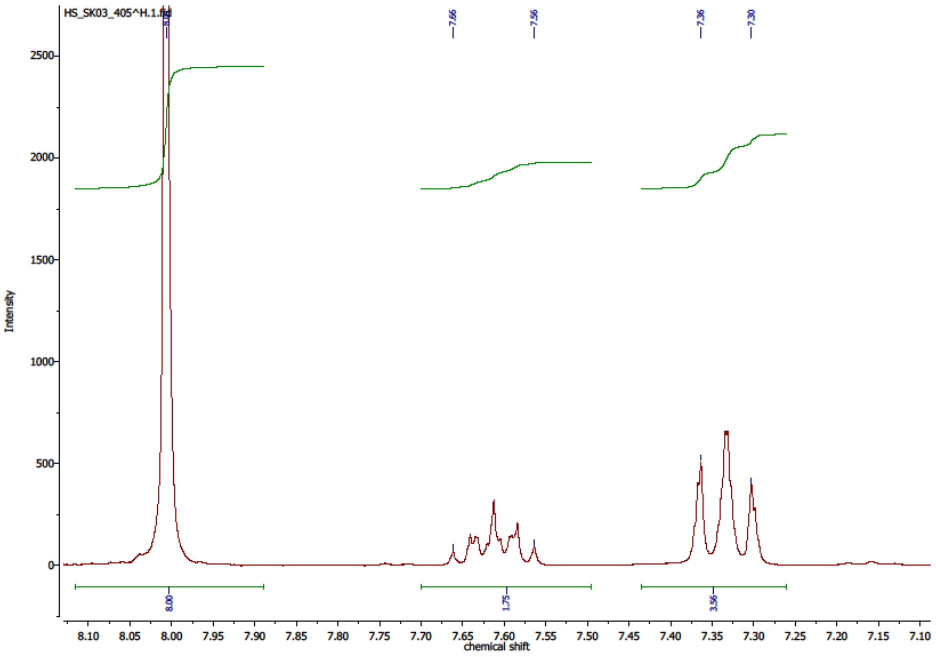

Figure S14. ^{1}H NMR spectrum of *tF*-AZB@DMOF-1 (system **3**) digested in 1 ml of DMSO-d_6 and 25 µl of DCl after irradiation with blue light (λ = 405 nm) for 15 min. The *E*/*Z* ratio was found to be 96% *E* isomer.

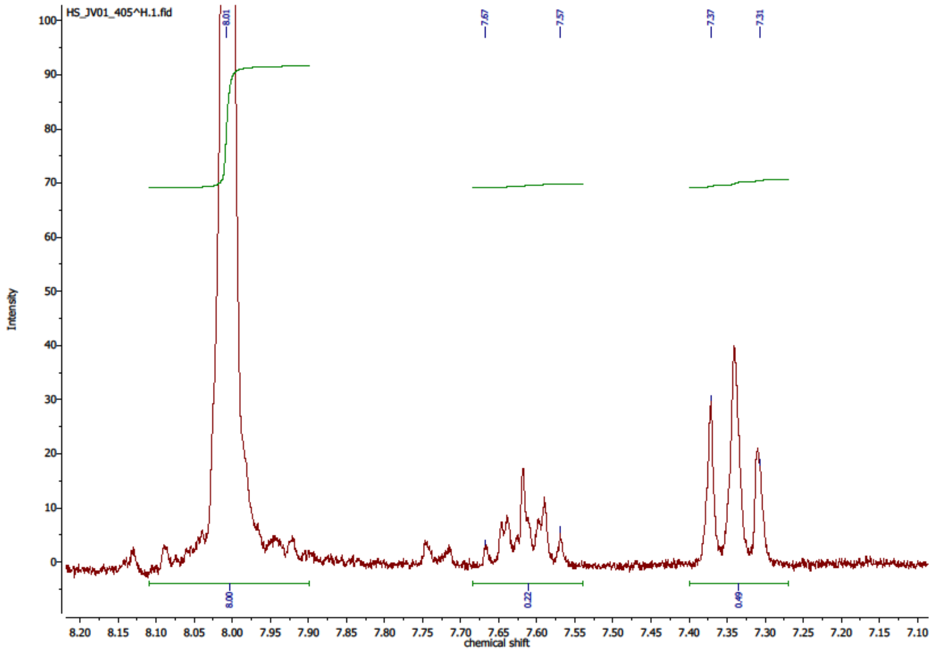

Figure S15. ^{1}H NMR spectrum of *tF*-AZB@DMOF-1 (system **4**) digested in 1 ml of DMSO-d_6 and 25 µl of DCl after irradiation with blue light (λ = 405 nm) for 15 min. The *E*/*Z* ratio was found to be 100% *E* isomer.

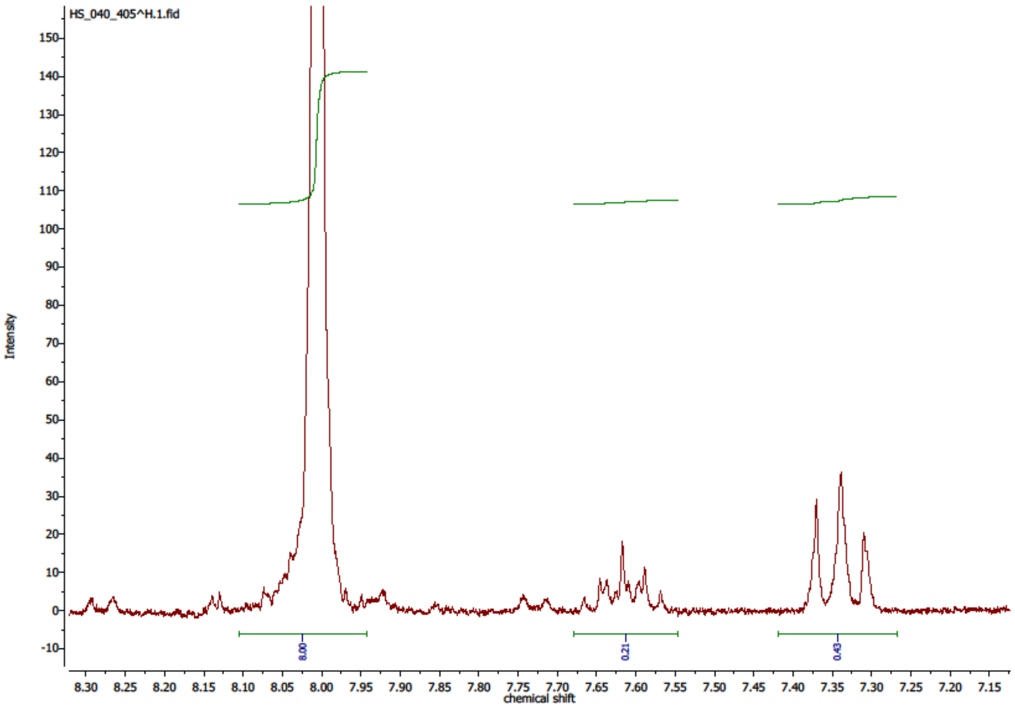

Figure S16. ^{1}H NMR spectrum of *tF*-AZB@DMOF-1 (system **5**) digested in 1 ml of DMSO-d_6 and 25 µl of DCl after irradiation with blue light (λ = 405 nm) for 15 min. The *E*/*Z* ratio was found to be 100% *E* isomer.

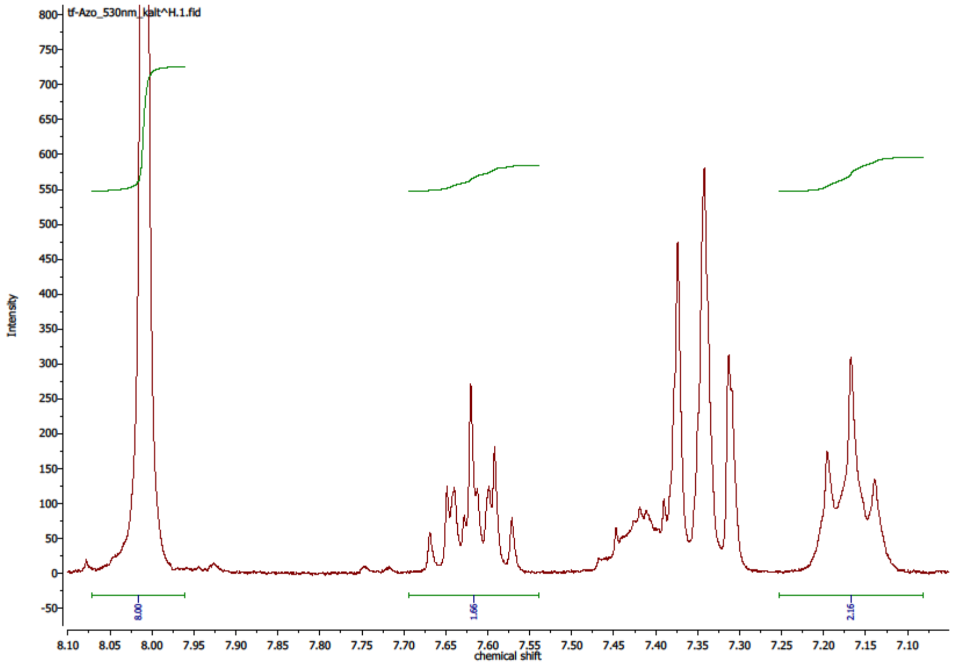

Figure S17. ^{1}H NMR spectrum of *tF*-AZB@DMOF-1 (system **1**) digested in 1 ml of DMSO-d_6 and 25 µl of DCl after irradiation with blue light (λ = 535 nm) for 15 min. The *E*/*Z* ratio was found to be 60.5% *E* isomer.

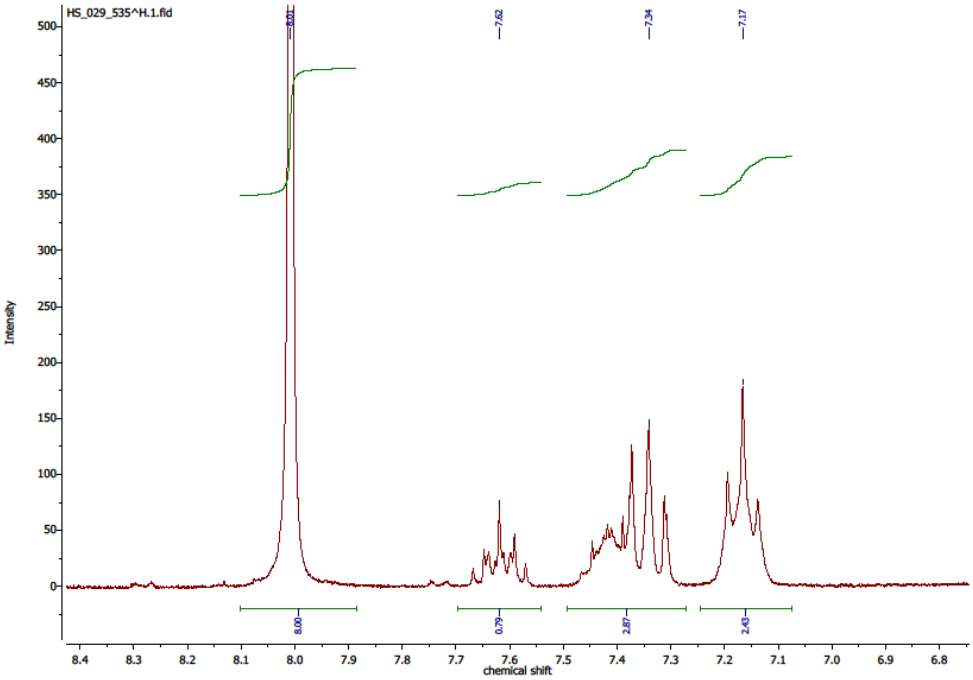

Figure S18. ^{1}H NMR spectrum of *tF*-AZB@DMOF-1 (system **2**) digested in 1 ml of DMSO-d_6 and 25 µl of DCl after irradiation with blue light (λ = 535 nm) for 15 min. The *E*/*Z* ratio was found to be 39.3% *E* isomer.

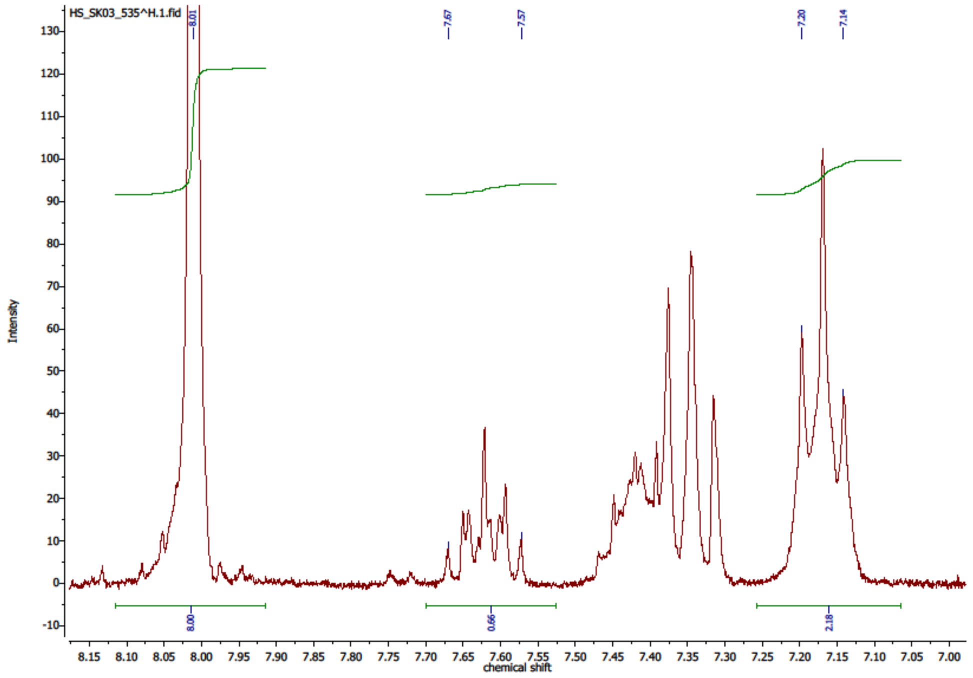

Figure S19. ^{1}H NMR spectrum of *tF*-AZB@DMOF-1 (system **3**) digested in 1 ml of DMSO-d_6 and 25 µl of DCl after irradiation with blue light (λ = 535 nm) for 15 min. The *E*/*Z* ratio was found to be 37.7% *E* isomer.

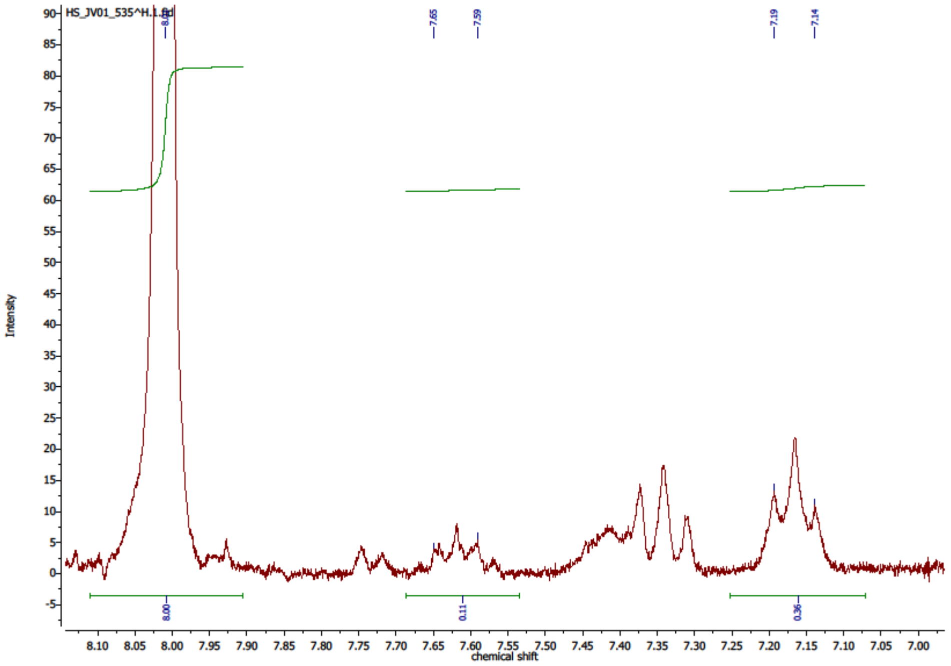

Figure S20. ^{1}H NMR spectrum of *tF*-AZB@DMOF-1 (system **4**) digested in 1 ml of DMSO-d_6 and 25 µl of DCl after irradiation with blue light (λ = 535 nm) for 15 min. The *E*/*Z* ratio was found to be 38% *E* isomer.

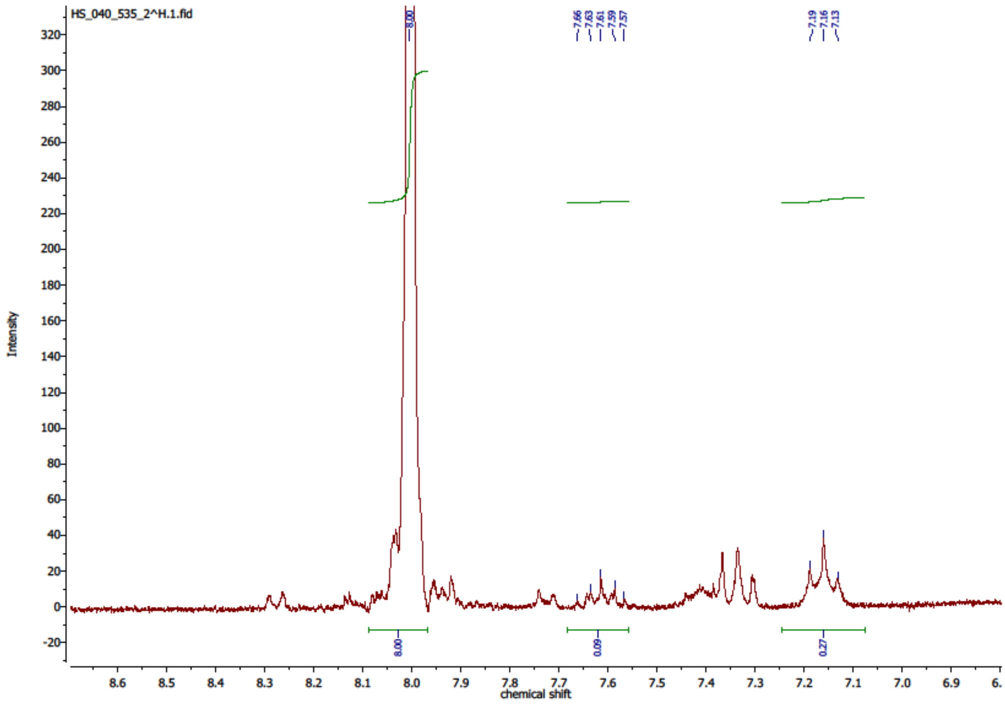

Figure S21. ^{1}H NMR spectrum of *tF*-AZB@DMOF-1 (system **5**) digested in 1 ml of DMSO-d_6 and 25 µl of DCl after irradiation with blue light (λ = 535 nm) for 15 min. The *E*/*Z* ratio was found to be 40% *E* isomer.

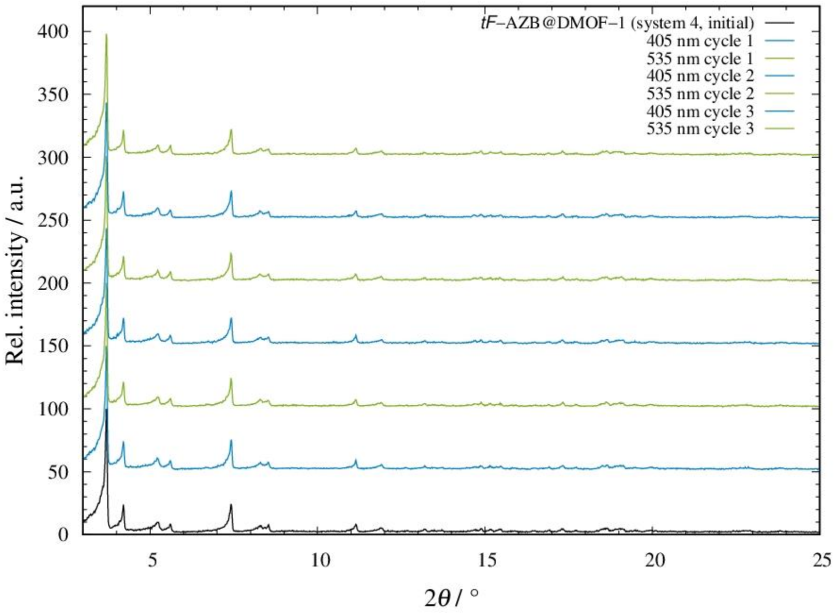

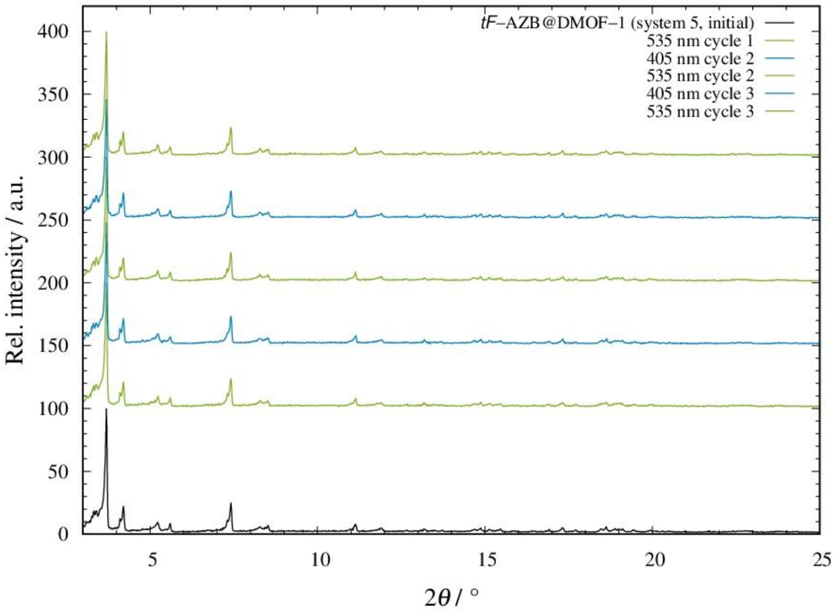

Figure S22. Diffraction patterns of system **4** (top) and system **5** (bottom), when non-irradiated (black line), irradiated with blue light (blue lines) and green light (green lines) with off-set. The diffraction patterns were measured at 298 K (*Stoe Stadi P*: λ = 0.7107 Å).

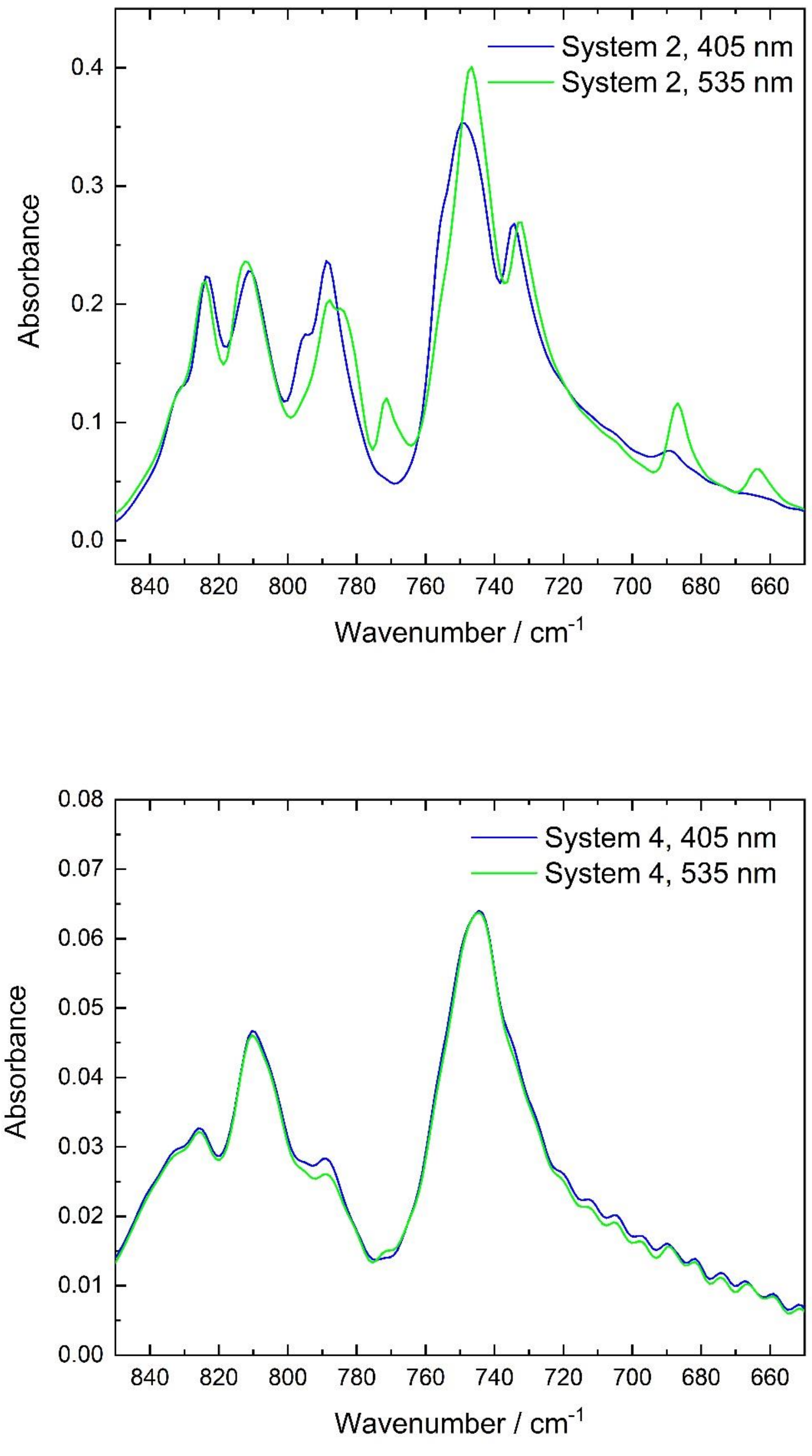

Figure S23. IR spectra of systems **2** (top) and system **4** (bottom), when irradiated with blue light (blue lines) and green light (green lines). Irradiation was carried out for 5 min under an argon atmosphere.

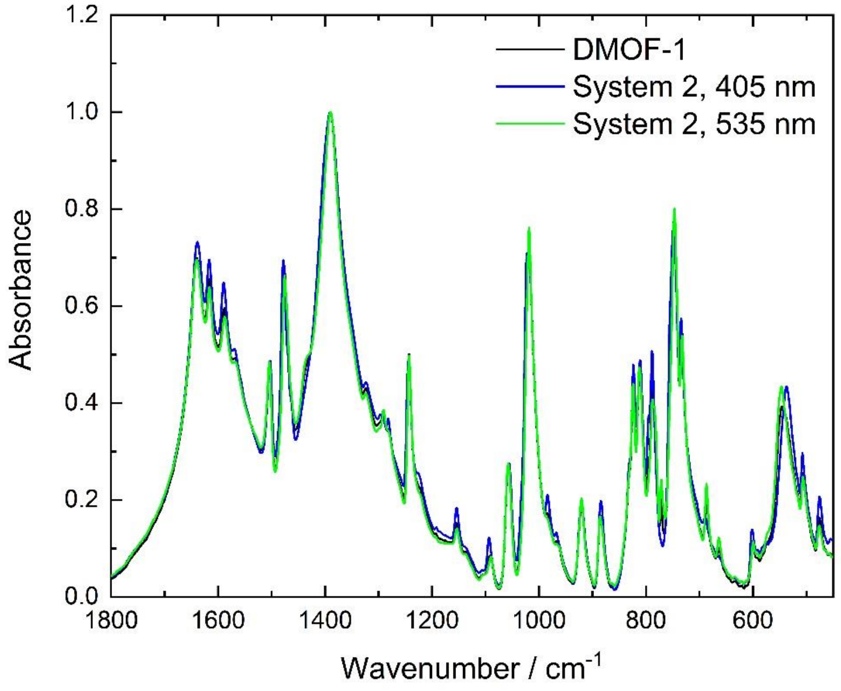

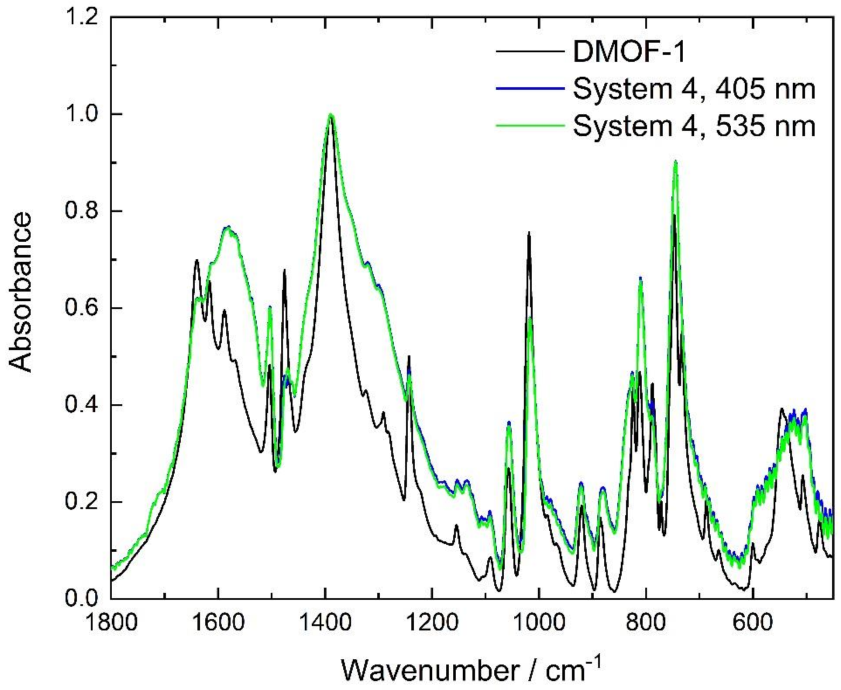

Figure S24. IR spectra of systems **2** (top) and system **4** (bottom), when irradiated with blue light (blue lines) and green light (green lines) compared to pure DMOF-1. Irradiation was carried out for 5 min under an argon atmosphere.

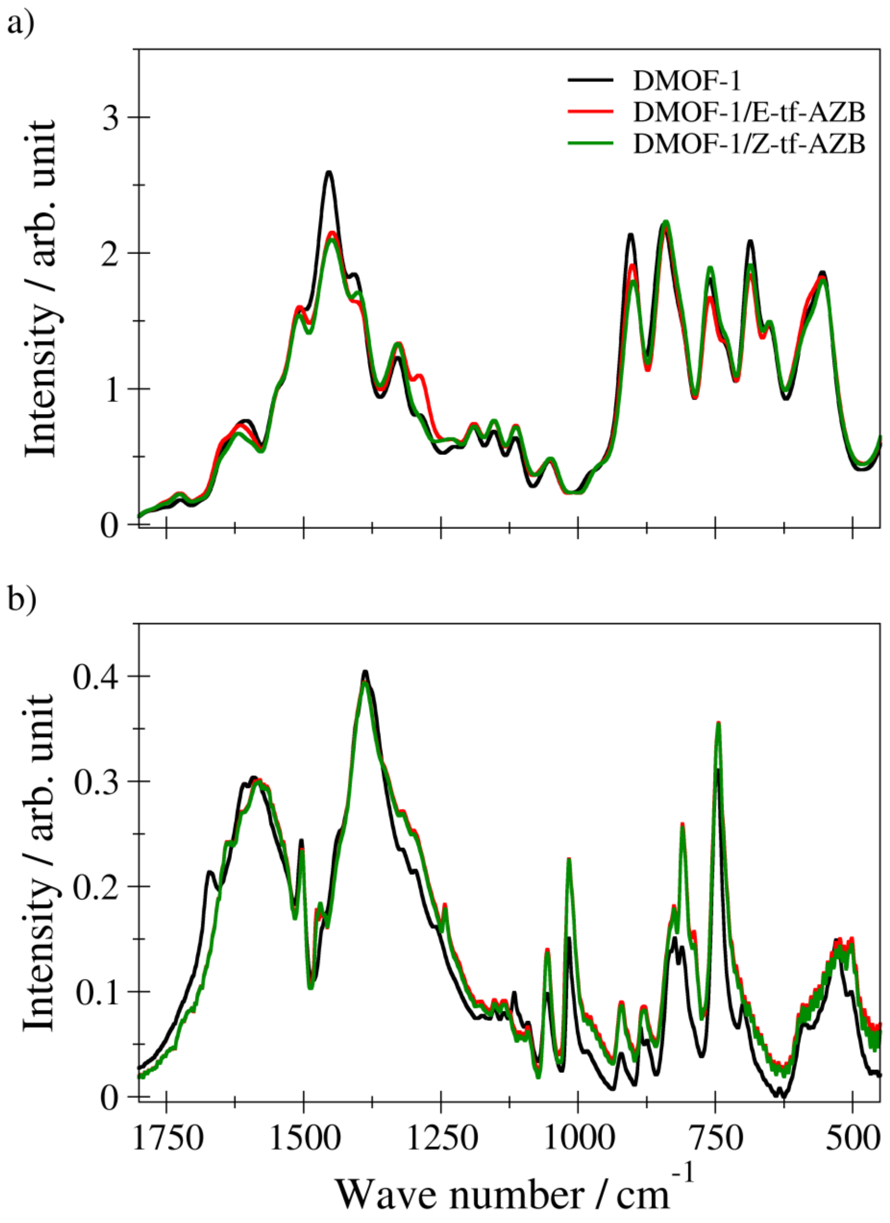

Figure S25. Comparison of a) the calculated IR spectra obtained from the SCC DFTB MD simulations against b) the experimental reference for DMOF-1 as well as the *E*/*Z*-*tF*-AZB@DMOF-1 complexes. Bands in the high energy range are not accessible in this case due to the rigid-body constraints applied to all X-H bonds.

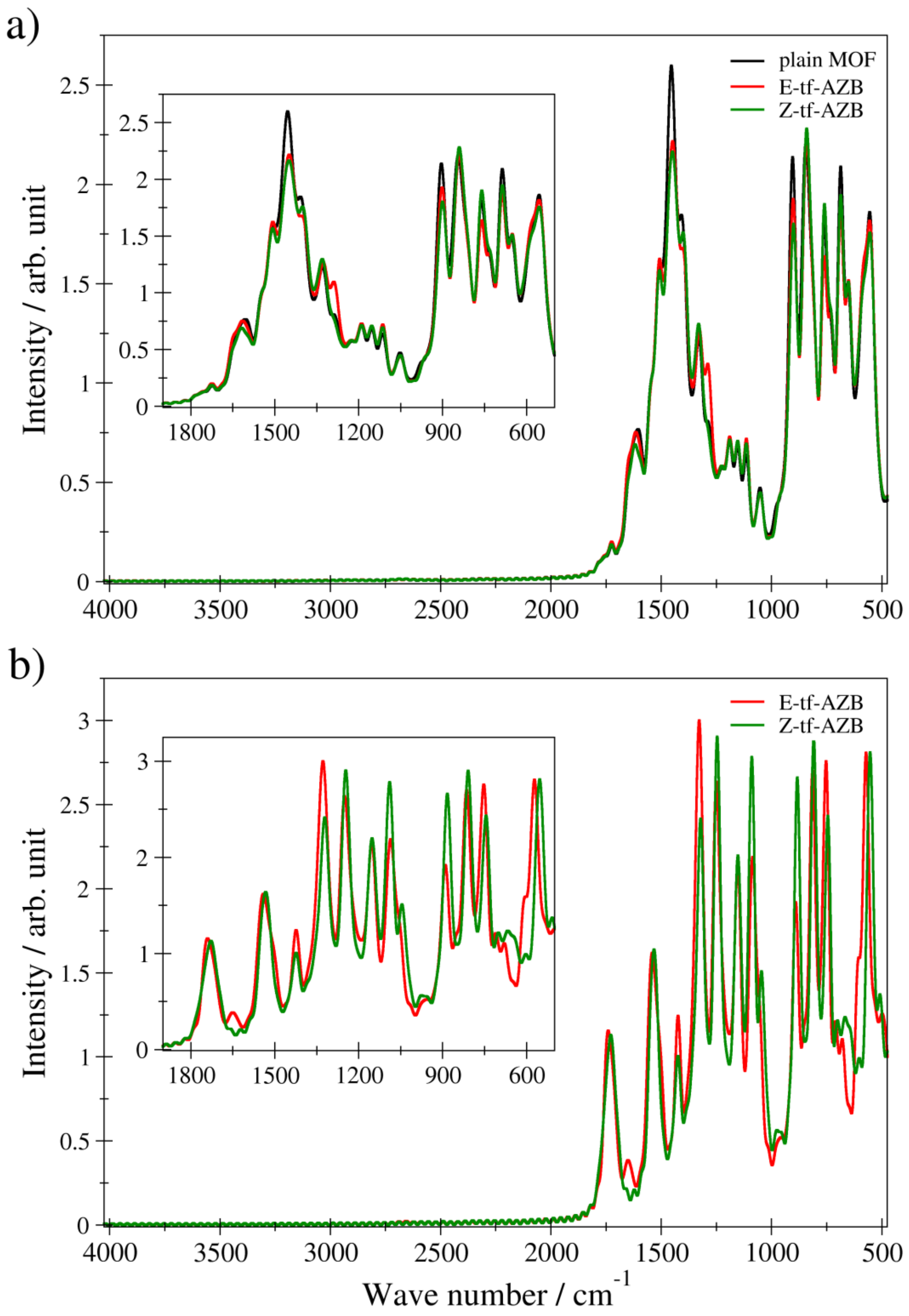

Figure S26. a) Calculated IR spectra determined from the SCC DFTB MD simulations for DMOF-1 as well as the *E*/*Z*-*tF*-AZB@DMOF-1 complexes. b) Calculated IR spectra obtained by considering only the contributions of the E/Z-tf-AZB molecules in DMOF-1.

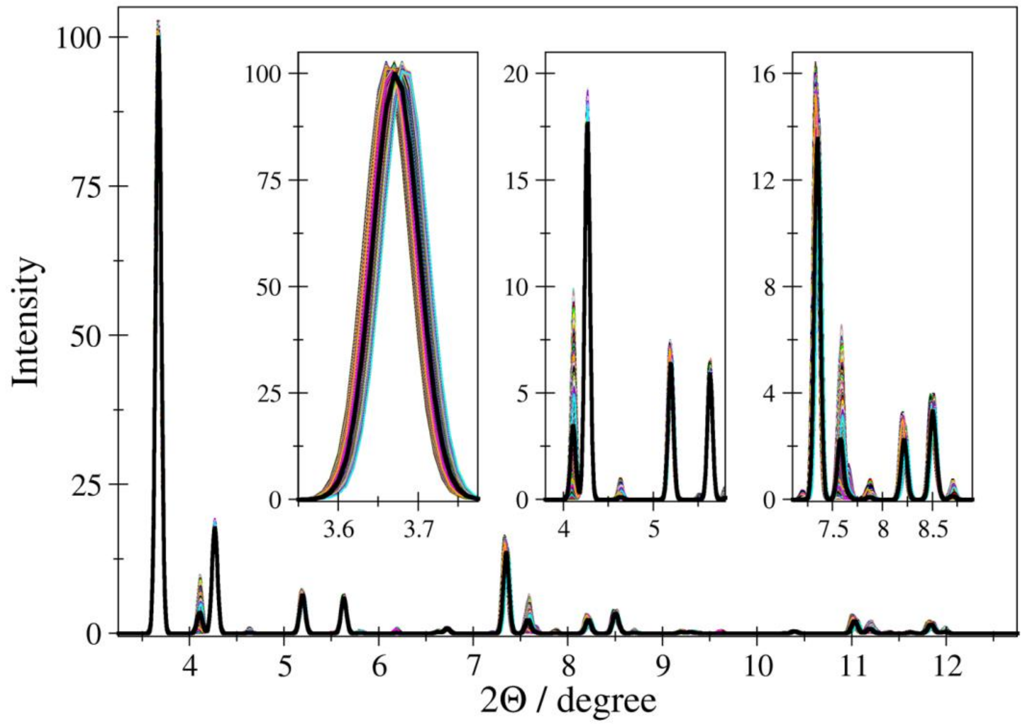

Figure S27. Average XRPD pattern (black) of DMOF-1 obtained by averaging 5000 individual patterns (color) computed for every 5th configuration of the MD sampling phase. All patterns were scaled with respect to the average XRPD pattern, which has been normalized to 100.

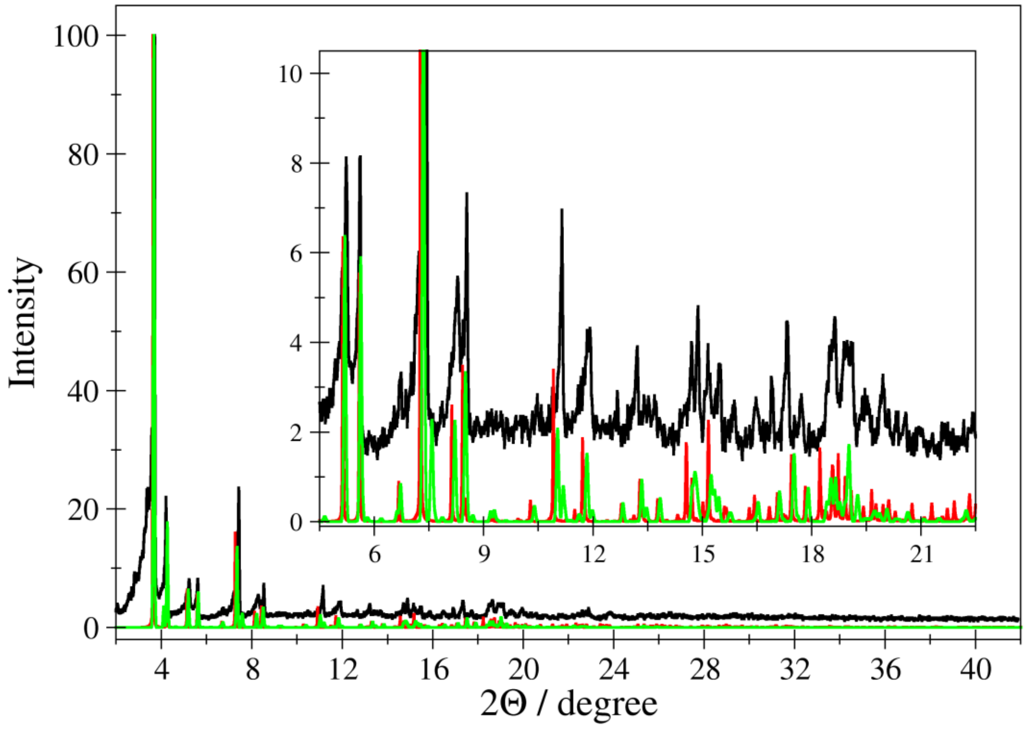

Figure S28. Comparison of the XRPD pattern for empty DMOF-1 obtained via a single minimum configuration (red) and thermal averaging over the MD trajectory (green) against the experimental reference (black). All patterns have been normalized to a maximum value of 100.

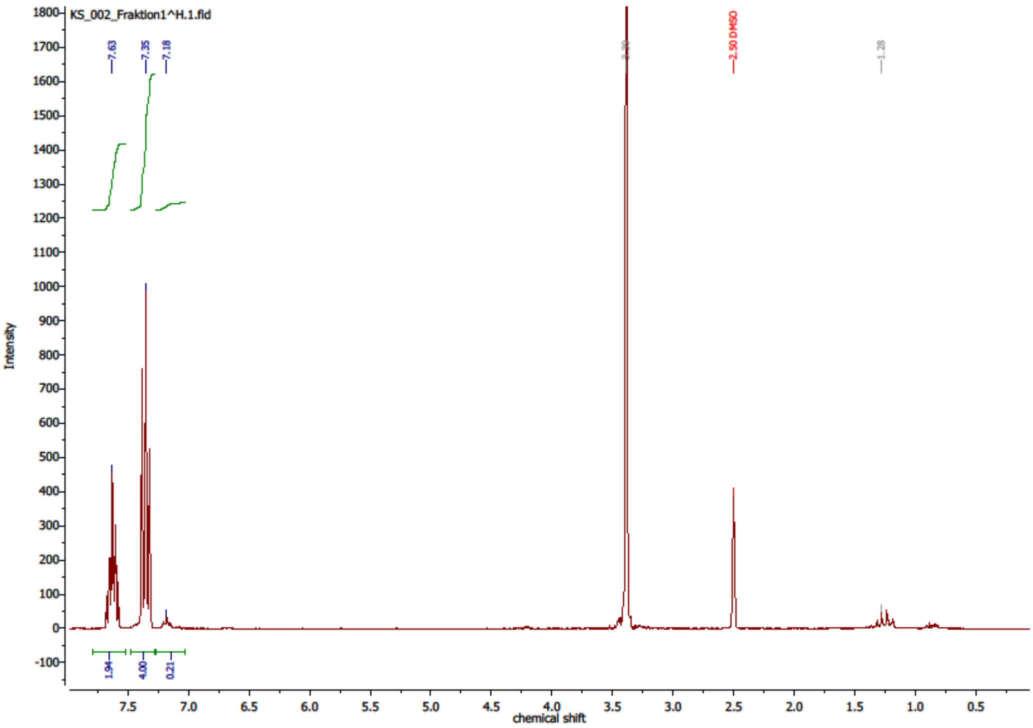

Figure S29. ^{1}H NMR spectrum of *tF*-AZB digested in DMSO-d_6. Impurities at <1.5 ppm originate from grease.

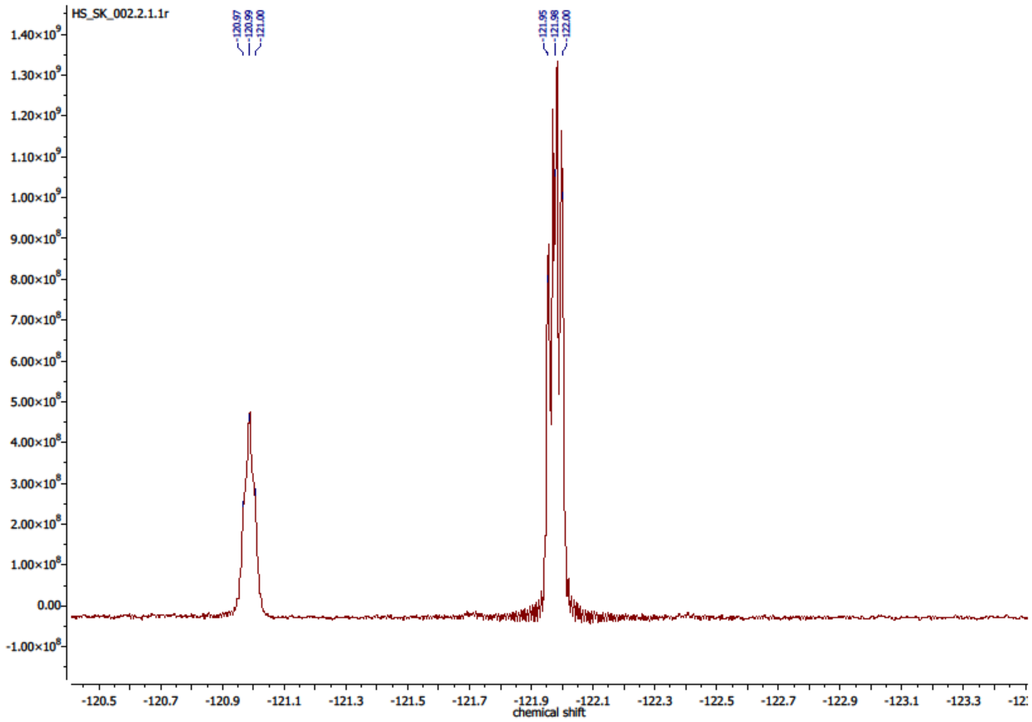

Figure S30. ^{19}F NMR spectrum of *tF*-AZB digested in DMSO-d_6.

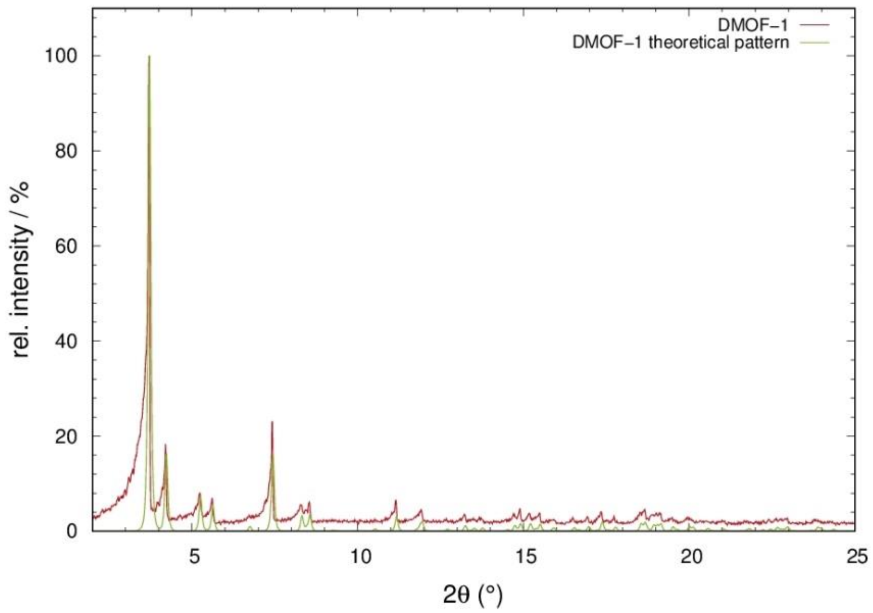

Figure S31. Diffraction pattern of unloaded DMOF-1 (red line) in comparison to the peak intensities and position calculated from theoretical data[1] (green line). The diffraction pattern was measured at 298 K (*Stoe Stadi P*: λ = 0.7093 Å).

References

[1] D. N. Dybtsev, H. Chun, K. Kim, *Angew. Chem. - Int. Ed.* **2004**, *43*, 5033–5036.

[2] I. M. Walton, J. M. Cox, J. A. Coppin, C. M. Linderman, D. G. (Dan) Patel, J. B. Benedict, H. Ren, G. Zhu, *Chem. Commun.* **2013**, *49*, 8012–8014.
